# Preparation of RNAs with non-canonical 5′ ends using novel di- and trinucleotide reagents for co-transcriptional capping

**DOI:** 10.3389/fmolb.2022.854170

**Published:** 2022-08-19

**Authors:** Anaïs Depaix, Ewa Grudzien-Nogalska, Bartlomiej Fedorczyk, Megerditch Kiledjian, Jacek Jemielity, Joanna Kowalska

**Affiliations:** ^1^ Division of Biophysics, Institute of Experimental Physics, Faculty of Physics, University of Warsaw, Warsaw, Poland; ^2^ Centre of New Technologies, University of Warsaw, Warsaw, Poland; ^3^ Department of Cell Biology and Neuroscience, Rutgers University, New York, NJ, United States

**Keywords:** NAD, FAD, UDP-glucose, RNA cap, *In vitro* transcription, dinucleotide, trinucleotide

## Abstract

Many eukaryotic and some bacterial RNAs are modified at the 5′ end by the addition of cap structures. In addition to the classic 7-methylguanosine 5′ cap in eukaryotic mRNA, several non-canonical caps have recently been identified, including NAD-linked, FAD-linked, and UDP-glucose-linked RNAs. However, studies of the biochemical properties of these caps are impaired by the limited access to *in vitro* transcribed RNA probes of high quality, as the typical capping efficiencies with NAD or FAD dinucleotides achieved in the presence of T7 polymerase rarely exceed 50%, and pyrimidine derivatives are not incorporated because of promoter sequence limitations. To address this issue, we developed a series of di- and trinucleotide capping reagents and *in vitro* transcription conditions to provide straightforward access to unconventionally capped RNAs with improved 5′-end homogeneity. We show that because of the transcription start site flexibility of T7 polymerase, R^1^ppApG-type structures (where R^1^ is either nicotinamide riboside or riboflavin) are efficiently incorporated into RNA during transcription from dsDNA templates containing both φ 6.5 and φ 2.5 promoters and enable high capping efficiencies (∼90%). Moreover, uridine-initiated RNAs are accessible by transcription from templates containing the φ 6.5 promoter performed in the presence of R^2^ppUpG-type initiating nucleotides (where R^2^ is a sugar or phosphate moiety). We successfully employed this strategy to obtain several nucleotide-sugar-capped and uncapped RNAs. The capping reagents developed herein provide easy access to chemical probes to elucidate the biological roles of non-canonical RNA 5′ capping.

## 1 Introduction

Currently, *in vitro* transcription catalyzed by bacterial or viral RNA polymerases is the only method that provides straightforward access to RNAs of any sequence and unlimited length (Milligan et al., 1987; Beckert and Masquida, 2011). *In vitro* transcribed (IVT) RNAs are not only invaluable research tools but have also recently emerged as a new class of vaccines and therapeutics (Fuller and Berglund, 2020; Pardi et al., 2020; Jia and Qian, 2021; Pascolo, 2021). Recent discoveries in the field of epigenetic RNA modifications have uncovered several novel regulatory mechanisms (Wang et al., 2019; Huang et al., 2020; Netzband and Pager, 2020; Zhao et al., 2020), which have created a demand for simple methods providing access to chemically modified RNAs (Abele et al., 2020; Mattay et al., 2021; Ren et al., 2021). One important class of modified RNAs is RNAs specifically derivatized at the 5′ end. The best and longest-known examples are eukaryotic mRNAs and snRNAs carrying 7-methylguanosine and 2,2,7-trimethylguanosine caps, respectively (Furuichi et al., 1975; Wei et al., 1975; Furuichi and Shatkin, 2000). However, many unconventionally capped RNAs have been recently discovered in both eukaryotic and prokaryotic cells, including RNA 5′-linked to NAD (NAD-RNA) (Chen et al., 2009; Cahová et al., 2015), FAD (FAD-RNA) (Wang et al., 2019), coenzyme-A (CoA-RNA) (Kowtoniuk et al., 2009), dinucleoside polyphosphates (Np_n_Ns-RNA) (Luciano et al., 2019; Hudecek et al., 2020), vitamins (thiamine-capped RNAs) (Mohler et al., 2020), or nucleotide sugars (Glc-ppRNA and N-AcGlc-ppRNA) (Julius and Yuzenkova, 2017; Wang et al., 2019; Wiedermannova et al., 2021). Access to these 5′-modified RNAs by IVT is often limited by the specificity and promoter sequences of RNA polymerases (Huang, 2003; Benoni et al., 2020).

The *in vitro* transcription reaction is typically performed by bacteriophage RNA polymerases (e.g., the T3, SP6, or T7 phage polymerase used in this study), using a mixture of four natural nucleoside 5′-triphosphates (NTPs) and a double-stranded dsDNA template (Beckert and Masquida, 2011). The DNA template must begin with an appropriate promoter sequence consisting of a polymerase recruitment site, followed by or overlapping with the transcription start site (TSS), and then the transcribed nucleotides. The polymerase initiates transcription when the 3′-OH of the first transcribed NTP, defined by the TSS, attacks the α-phosphate of the subsequent NTP, yielding a 5′-triphosphate of the nascent RNA chain. If a dinucleotide analog of the Np_n_N’ (N′ is the nucleoside defined by the TSS) structure is added to the transcription mixture, it can serve as an alternative transcription initiator, leading to the corresponding capped RNA (Np_n_N′-RNA). Two common promoters used for transcription by T7 polymerase are φ 2.5 (...T^-1^A^+1^G^+2^G^+3^) and φ 6.5 (...A^-1^G^+1^G^+2^G^+3^; wherein +1 denotes the position of the standard TSS), which trigger initiation with ATP and GTP, respectively. Transcription from templates containing these promoters provides access to purine-initiated uncapped RNAs and RNAs capped with cap structures containing G (e.g., m^7^GpppG) or A (e.g., NAD) as the 5′ terminal nucleotide, thereby producing m^7^GpppG-capped RNAs or NAD-capped RNAs, respectively (Huang, 2003; Grudzien-Nogalska et al., 2007; Julius et al., 2020). However, the preparation of RNAs initiated by pyrimidine nucleotides or modified purine nucleotides is not possible using dinucleotide analogs and standard promoters, because of their incompatibility with TSS in the template or the structural requirements of the RNA polymerase. Furthermore, dinucleotide capping reagents rarely trigger the complete capping of RNA. In the case of NAD-linked and FAD-linked RNAs, approximately 50% of produced RNA may remain uncapped, even under optimized conditions (Huang, 2003). To overcome some of these disadvantages, we and others have recently developed trinucleotide analogs of m^7^G caps and NAD caps (of Np_n_N′pG general structure), which provided higher RNA capping efficiencies than dinucleotides and enabled more structural variability within the position of the first transcribed nucleotide (Ishikawa et al., 2009; Sikorski et al., 2020; Depaix et al., 2021). Another class of dinucleotide analogs such as 5′-Dy-ApG has also been used as transcription initiators providing access to 5'-fluorescently labeled RNAs (Wang et al., 2013).

Here, we generalize this approach, demonstrating that it can be harnessed to obtain a variety of unconventional 5′-capped RNAs. We designed and chemically synthesized trinucleotide analogs of FAD and nucleotidesugar “caps,” as well as dinucleotide analogs of pyrimidine-initiated uncapped RNA, which are compatible with transcription from templates containing φ 2.5 and/or φ 6.5 T7 RNA polymerase promoters. We studied the incorporation of these compounds by T7 RNA polymerase, along with the corresponding dinucleotides and previously synthesized NADpG, to establish conditions providing optimal capping efficiency. We determined the transcription conditions providing access to efficiently capped NAD-RNA, FAD-RNAs, and GlcppU-RNAs and identified issues that may impair the analysis of capping efficiencies for unconventionally capped RNAs. We also addressed the problem of generating uncapped RNAs initiated with pyrimidine nucleotides using polymerase T7 by employing pppUpG as a transcription initiator to yield pppU-RNAs.

## 2 Materials and methods

### 2.1 General information for the chemical synthesis

Starting materials, solvents and chemical reagents were acquired from commercial sources. Glucose phosphates and pyrophosphate were purchased as sodium salts and converted into triethylammonium salts before their use in synthesis. For that purpose, the solutions of sodium salts were passed through Dowex-50 W-X8 cationite column, which was pre-conditioned by washing with 1 M sodium hydroxide in deionized water, followed by deionized water, 5% hydrochloric acid in deionized water, deionized water, triethylamine, and finally, deionized water. The collected eluate was evaporated to dryness under reduced pressure and the residue was dried under vacuum over P_2_O_5_.

Solid-supported syntheses were performed using ÄKTA Oligopilot plus 10 synthesizer (GE Healthcare).

Analytical RP HPLC was performed on Agilent apparatus using GEMINI LC-18-T HPLC column (4.6 mm × 250 mm, 5 μm, flow rate 1.3 ml min^1^). A linear gradient of 0–50% methanol in buffer A (ammonium acetate buffer, pH 5.9, 0.05 M) over 30 min was typically used. All the nucleotides were UV-detected at 254 nm.

Isolation of the nucleotides was performed by ion-exchange chromatography on a DEAE Sephadex A-25 (HCO_3_^-^ form) column. To this end, the column was loaded with the quenched reaction mixture and washed thoroughly with water until the eluate did not precipitate with AgNO_3_ solution, in order to remove solvents and unbound reagents. Nucleotides were then eluted with the use of a linear gradient of triethylammonium bicarbonate buffer (TEAB) in deionized water. Collected fractions were analyzed spectrophotometrically at 260 nm and if necessary by RP-HPLC. Fractions containing the desired product were combined. After concentration under reduced pressure with repeated additions of ethanol (96%) followed by precipitation in acetonitrile (to decompose TEAB and to remove residual water, respectively), compounds were isolated as triethylammonium salts.

All final products were additionally purified on a semi-preparative RP-HPLC column (Discovery RP Amide C-16 250 mm × 21.2 mm, 5 μm, flow rate 5.0 ml min^-1^) using UV detection at 254 nm. A linear gradient of acetonitrile (from 0 to 50% of ACN for 60 min) in buffer A (0.05 M ammonium acetate, pH 5.9) was applied except for uridine derivatives which were purified using an isocratic buffer A elution. After repeated freeze-drying of the collected fractions, the products were isolated as ammonium (NH_4_^+^) salts.

Yields were calculated on the basis of UV absorbance of the aqueous solutions of isolated products and corresponding starting materials. Absorbance measurements were performed after diluting an aliquot of the stock solution of the compound in a 0.1 M phosphate buffer at 260 nm (pH 7.0). The following absorption coefficients (ε_260_, ml/mmol/cm) were used to determine compound concentrations: 17400 for NAD; 29480 for NADpG; 37000 For FAD, 49080 for FADpG and 19,566 for nucleotides containing U and G nucleobases.

The structures and homogeneity of all final compounds were confirmed by RP-HPLC, high-resolution mass spectrometry using electrospray ionization (HRMS-ESI) and ^1^H and ^31^P NMR spectroscopy, unless stated otherwise. Mass spectra were recorded with LTQ Orbitrap Velos (Thermo Scientific) spectrometer. NMR spectra were recorded at 25°C with a BRUKER AVANCE III HD spectrometer at 500 MHz (^1^H NMR) and 202 MHz (^31^P NMR), or at 400 and 162 MHz, respectively, on a Varian UNITY spectrometer. ^1^H NMR chemical shifts were calibrated to sodium 3-trimethylsilyl-[2,2,3,3-S5,D4]propionate (TSP) in D_2_O and for ^31^P NMR to H_3_PO_4_ (20%) in D_2_O as an external standard. Signal assignments and identification were based on COSY spectra analysis. The raw NMR spectroscopic data were processed by MestReNova v 12.0.2-20,910 Software.

### 2.2 Synthesis of capping reagents

#### 2.2.1 General procedure A: Synthesis of dinucleotide 5′-monophosphates (pNpG)

The syntheses were performed using an automatic synthesizer ÄKTA Oligopilot plus 10 on a high-loaded polystyrene support (Primer Support 5G Ribo G 300, GE Healthcare) on a 50 μmol scale (81.2 mg, 308 μmol/g) using standard phosphoramidite chemistry. In the coupling steps, 5-(benzylthio)-1-*H*-tetrazole (0.30 M in acetonitrile) and 0.2 M acetonitrile solution of an appropriate TBDMS-protected adenosine or uridine phosphoramidite (step 1) and bis (cyanoethyl)- *N*,*N*- diisopropylphosphoramidite (*ChemGenes®*) (step 2) were recirculated through the column for 15 min. The detritylation step was performed using 3% (v/v) dichloroacetic acid in toluene, the oxidation was performed with 0.05 M iodine in pyridine. The capping step was performed using 20% (v/v) N-methylimidazole in acetonitrile as Cap A and a mixture of 40% (v/v) acetic anhydride and 40% (v/v) pyridine in acetonitrile as Cap B. The 2-cyanoethyl groups were removed after the last cycle by passing 20% (v/v) diethylamine in acetonitrile through the column. The solid support was finally dried with argon, transferred to a falcon tube and the dinucleotide was cleaved from the support at 37°C for 1 h using 5 ml of AMA (Ammonium hydroxide/Methyl amine, 1/1). The suspension was filtered, evaporated to dryness, freeze-dried from water and then resuspended in 100 μl of DMSO before the addition of 125 μl of triethylammonium trihydrofluoride (TEA·3HF), in order to remove the TBDMS protecting groups. The resulting solution was incubated at 65°C for 2 h and diluted with 10 ml of 0.2 M NaHCO_3_ to adjust the pH to 7. The crude product was purified by DEAE Sephadex using a linear gradient of TEAB (0–0.9 M) and isolated as a triethylammonium salt. Fractions containing the dinucleotide were combined and evaporated to dryness.

##### 2.2.1.1 pApG

The synthesis was performed following the general procedure **A** using *N*-PAC-5′-*O*-DMT-2′-*O*-TBDMS-adenosine 3′-*O*-(cyanoethyl-*N*,*N*-diisopropylphosphoramidite) (*ChemGenes®*) (154.0 mg, 165 μmol, 2.5 equiv., 0.2 M in acetonitrile). This procedure allowed to obtain 3’-(guanyl-5′-yl)-adenosine 5′-monophosphate (pApG, 30.7 μmol, 61% yield).

$${\text{HPLC: }t}_{r}\;=\;8\text{.}67\text{ min}$$

HR-MS (ESI, m/z)**:** Calculated for C_20_H_25_N_10_O_14_P_2_^-^ [(M-H)^-^]: 691.10324; Found: 691.10392.

##### 2.2.1.2 pUpG

The synthesis was performed following the general procedure **A** using 5’-*O*-DMT-2′-*O*-TBDMS-uridine 3′-*O*-(cyanoethyl-*N*,*N*-diisopropylphosphoramidite) (*ChemGenes®*) (142.1 mg, 165 μmol, 2.5 equiv., 0.2 M in acetonitrile). This procedure allowed to obtain 3’-(guanyl-5′-yl)-uridine 5′-monophosphate (pUpG, 31.9 μmol, 64% yield).

$${\text{HPLC: }t}_{r}\;=\;5\text{.}87\text{ min}$$

HR-MS(ESI, m/z): Calculated for C_19_H_24_N_7_O_16_P_2_^-^([M-H]^-^): 668.07602; Found: 668.07627.

#### 2.2.2 General procedure B: Activation of mono- or dinucleotides

The desired mono- or dinucleotide was suspended in DMSO (C = 0.1 M), followed by the addition of imidazole, 2,2′-dithiodipyridine, triethylamine, and triphenylphosphine. The reaction was stirred at r.t. Until a complete conversion was determined by RP-HPLC analysis. The reaction was quenched by the addition of a cold solution of sodium perchlorate (3 equiv.) in acetone (10× V_reaction_). The resulting precipitate was washed with cold acetone and centrifuged until the supernatant was clear. The resulting powder was then dried under vacuum for few hours, affording the imidazolide (Im) activated nucleotide (ImpNpG or NMP-Im).

##### 2.2.2.1 ImpApG

The synthesis was performed following the general procedure **B** using pApG (749 mOD, 30.7 µmol), imidazole (33.4 mg, 0.491 mmol, 16 equiv.), 2,2′-dithiodipyridine (40.5 mg, 0.184 mmol, 6 equiv.), triethylamine (12.9 µl, 0.092 mmol, 3 equiv.) and triphenylphosphine (48.3 mg, 0.184 mmol, 6 equiv.) in DMSO (0.31 ml). The reaction was stirred at r.t. for 48 h. After precipitation, ImpApG was obtained as a sodium salt (30.0 μmol, 98% yield).

$${\text{HPLC: }t}_{r}\;=\;11\text{.}71\text{ min}$$

##### 2.2.2.2 UMP-Im

The synthesis was performed following the general procedure **B** using commercial sodium salt UMP transformed in triethylammonium salt (889 mOD, 0.092 mmol), imidazole (62.6 mg, 0.092 mmol, 10 equiv.) 2,2′-dithiodipyridine (60.7 mg, 0.276 mmol, 3 equiv.), triethylamine (38.7 µl, 0.276 mmol, 3 equiv.) and triphenylphosphine (72.3 mg, 0.276 mmol, 3 equiv.) in DMSO (0.9 ml). The reaction was stirred at r.t. for 1 h. After precipitation, UMP-Im was obtained as sodium salt and used directly in further coupling step.

##### 2.2.2.3 ImpUpG

The synthesis was performed following the general procedure **B** using pUpG (0.029 mmol), imidazole (31.3 mg, 0.461 mmol, 16 equiv.) 2,2′-dithiodipyridine (38.0 mg, 0.173 mmol, 6 equiv.), triethylamine (12.1 µl, 0.086 mmol, 3 equiv.) and triphenylphosphine (45.3 mg, 0.173 mmol, 6 equiv.) in DMSO (0.3 ml). The reaction was stirred at r.t. for 16 h. After precipitation, ImpUpG was obtained as sodium salt and used directly in further coupling step.

#### 2.2.3 General procedure C: coupling reaction between phosphate nucleophiles and imidazolide derivatives

The nucleotide 5′-phosphorimidazolide (1 equiv.) was dissolved in DMSO or DMF. Then, zinc or magnesium chloride (3–8 equiv.) and the appropriate phosphate nucleophile (1–3 equiv.) were added. The reaction was mixed at r.t and the progress was monitored by RP HPLC. When a complete conversion of the starting material was observed, the reaction was quenched by dilution with a solution of equimolar disodium EDTA in 10 volumes of water and adjusted to pH 7 with sodium bicarbonate. The purification was performed by ion exchange chromatography (DEAE Sephadex) using a linear gradient of TEAB (from 0 to stipulated concentration), followed by a semi-preparative RP HPLC.

NADpG (1) was synthesized as described previously (Mlynarska-Cieslak et al., 2018).

##### 2.2.3.1 FADpG (2)

The synthesis was performed following the general coupling procedure **C** using ImpApG, (741 mOD, 0.030 mmol), flavine mononucleotide as triethylammonium salt (36.2 mg, 0.033 mmol, 1.5 equiv.) and zinc chloride (32.7 mg, 0.240 mmol, 8 equiv.,) in DMSO (0.75 ml). The reaction was stirred at r.t. for 16 h. After Sephadex (0.9 M TEAB) and RP-HPLC purifications, the expected product FADpG was obtained as ammonium salt (5.4 μmol, 18% yield).

$${\text{HPLC: }t}_{r}\;=\;10\text{.}8\text{ min}$$

^1^H NMR (400 MHz, D_2_O, 25°C): *δ* ^1^H NMR (400 MHz, D_2_O, 25C) 8.39 (s, 1Har), 8.02 (s, 1H), 7.79 (s, 1H, Har), 7.59 (s, 1H, Ph), 7.57 (s, 1H, Ph), 5.86 (d, *J* = 5.4 Hz, 1H, H1′a), 5.67 (d, *J* = 5.4 Hz, 1H, H1′b), 4.99–4.89 (m, 1H), 4.78–4.71 (m –overlap. solvent, 2H), 4.71–4.65 (m, 1H, H2′a), 4.60 (t, *J* = 5.4 Hz, 1H, H2′b), 4.44–4.40 (m, 1H, H3′b), 4.39–4.35 (m, 1H), 4.33–4.19 (m, 4H), 4.19–4.03 (m, 3H), 3.94 (dd, *J* = 7.3, 4.7 Hz, 1H), 2.36 (s, 3H, CH_3_), 2.28 (s, 3H, CH_3_), 2.00–1.96 (m, 1H). ^31^P NMR (162 MHz, D_2_O, 25°C)**:** δ -0.80 (s, 1P, A-P-G), -10.44 (d, 1P, Pα), -11.36 (d, 1P, Pβ). HR-MS (ESI, *m/z*): Calculated for C_37_H_44_N_14_O_22_P_3_^-^ ([M-H]^-^): 1129.19729; Found: 1129.19843.

##### 2.2.3.2 Uridine 5′-diphosphate *N*-acetylglucosamine, NAcGlcppU

The synthesis was performed following the general procedure **C** using UMP-Im (72 mOD, 7.5 µmol), *N*-acetylglucosamine-1-phosphate (NAcGlcp) as triethylammonium salt (7.5 µmol, 1 equiv.) and magnesium chloride (1.40 mg, 14.7 µmol, 2.8 equiv.) in DMF (0.064 ml). The reaction was stirred at r.t. for 2 h. After Sephadex (1 M TEAB) and RP-HPLC purifications, the expected product NAcGlcppU was obtained as ammonium salt (5.97 µmol, 80% yield).

$${\text{HPLC: }t}_{r}\;=\;1\text{.}78\text{ min}$$

HRMS (ESI, m/z): Calculated for C_17_H_26_N_3_O_17_P_2_^-^ [(M-H)^-^]: 606.07429; Found: 606.07455.

##### 2.2.3.3 3’-(guanyl-5′-yl)-uridine 5′-diphosphate glucose, GlcppUpG (3)

The synthesis was performed following the general procedure **C** using ImpUpG (0.015 mmol), glucose-1-phosphate as triethylammonium salt (8.6 mg, 0.024 mmol, 1.6 equiv.) and magnesium chloride (6.2 mg, 0.065 mmol, 2.7 equiv.) in DMF (0.2 ml). The reaction was stirred at r.t. for 30 h. After Sephadex (1 M TEAB) and RP-HPLC purifications, the expected product GlcppUpG was obtained as ammonium salt (7.7 μmol, 52% yield).

^1^H NMR (500 MHz, D_2_O, 25°C): *δ* 8.04 (s, 1H, H8-G), 7.86 (d, *J* = 8.1 Hz, 1H, H6-U), 5.93 (d, *J* = 8.1 Hz, 1H, H5-U), 5.89 (dd, *J* = 7.7, 6.1 Hz, 2H, H1′a+b), 5.63–5.58 (m, 1H, H1′Glc), 4.86–4.81 (m, 2H, H2′a), 4.70–4.60 (m, 1H), 4.54–4.46 (m, 1H, H3′a), 4.41–4.30 (m, 3H, H2′b), 4.24–4.07 (m, 4H, H3′b), 3.93–3.82 (m, 2H), 3.82–3.73 (m, 2H, H3′Glc), 3.53 (dt, *J* = 9.7, 3.1 Hz,1H, H2′Glc), 3.45 (t, *J* = 9.7 Hz, 1H, H4′Glc). ^31^P NMR (203 MHz, D_2_O, 25°C): *δ* -0.70 (s, U-P-G), -11.35 (d, P_α_), -12.76 (d, P_β_). HR-MS (ESI, *m/z*): Calculated for C_25_H_35_N_7_O_24_P_3_^-^ ([M-H]^-^): 910.09518; Found: 910.09497.

##### 2.2.3.4 3’-(guanyl-5′-yl)-uridine 5′-diphosphate *N*-acetylglucosamine, NAcGlcppUpG (4)

The synthesis was performed following the general procedure **C** using ImpUpG (11.3 mg, 0.015 mmol), *N*-Acetylglucosamine-1-phosphate as triethylammonium salt (0.015 mmol, 1 equiv.) and magnesium chloride (2.9 mg, 0.112 mmol, 3 equiv.) in DMF (0.13 ml). The reaction was stirred at r.t. for 48 h. After Sephadex (1 M TEAB) and RP-HPLC purifications, the expected product NAcGlcppUpG was obtained as ammonium salt (7.4 μmol, 49% yield).

$${\text{HPLC: }t}_{r}\;=\;5\text{.}38\text{ min}$$

^1^H NMR (400 MHz, D_2_O, 25°C): *δ* 8.01 (s, 1H, H8-G), 7.86 (d, *J* = 8.1 Hz, 1H, H6-U), 5.91 (d, *J* = 8.1 Hz, 1H, H5-U), 5.88 (dd, *J* = 5.7, 5.7 Hz, 2H, H1′a, H1′b), 5.51 (dd, *J* = 7.2, 3.3 Hz, 1H, H1′Glc), 4.83 (d, *J* = 7.6 Hz, 1H, H2′a), 4.66–4.60 (m, 1H), 4.53–4.48 (m, 1H, H3′a), 4.41–4.30 (m, 2H, H2′b), 4.22–4.08 (m, 3H), 4.02–3.90 (m, 2H, H2′Glc), 3.89–3.76 (m, 2H), 3.54 (dd, *J* = 10.1, 9.1 Hz, 1H), 2.06 (s, 3H, NAc). ^31^P NMR (162 MHz, D_2_O, 25°C): δ -0.78 (s, U-P-G), -11.59 (d, Pα), -13.09 (d, Pβ). HR-MS (ESI, *m/z*): Calculated for C_27_H_38_N_8_O_24_P_3_^-^ [(M-H)^-^]: 951.12173; Found: 951.12264.

##### 2.2.3.5 3’-(guanyl-5′-yl)-uridine 5′-triphosphate, pppUpG (5)

The synthesis was performed following the general procedure **C** using ImpUpG (718 mOD, 0.014 mmol), pyrophosphate as triethylammonium salt (16.0 mg, 0.042 mmol, 3 equiv.) and zinc chloride (15.2 mg, 0.112 mmol, 8 equiv.) in DMF (0.3 ml). The reaction was stirred at r.t. for 1 h. After Sephadex (1.2 M TEAB) and RP-HPLC purifications, the expected product pppUpG was obtained as ammonium salt (4.3 μmol, 31% yield).

$${\text{HPLC: t}}_{\text{r}}\;=\;3\text{.}69\text{ min}$$

^1^H NMR (400 MHz, D_2_O, 25°C): *δ* 7.99 (s, 1H, H8-G), 7.86 (d, *J* = 8.2 Hz, 1H, H5-U), 5.95 (d, *J* = 8.2 Hz, 1H, H6-U), 5.88 (d, *J* = 5.7 Hz, 1H, H1′U), 5.86 (d, *J* = 6.4 Hz, 1H, H1′G), 4.90–4.83 (m, 1H, H2′U), 4.67–4.62 (m, 1H), 4.50 (dd, *J* = 5.3, 3.9 Hz, 1H), 4.40–4.29 (m, 3H, H2′G), 4.23–4.05 (m, 4H). ^31^P NMR (162 MHz, D_2_O, 25°C): *δ* -0.86 (s, P_α_), -11.46 (d, P_γ_), -22.65 (t, P_β_). HR-MS (ESI, *m/z*): Calculated for C_19_H_26_N_7_O_22_P_4_^-^ [(M-H)^-^]: 828.00869; Found: 828.00929.

### 2.3 RNA synthesis and analysis

#### 2.3.1 *In vitro* transcription

NAD-capped RNAs were generated on the template of annealed oligonucleotides, which contained either a T7 A φ 2.5 promoter sequence (CAG​TAA​TAC​GAC​TCA​CTA​TT) followed by a 35-nt-long sequence (AGG GAA​GCG​GGC​ATG​CGG​CCA​GCC​ATA​GCC​GAT​CA), or G φ 6.5 promoter sequence (CAG​TAA​TAC​GAC​TCA​CTA​TA) followed by a 35-nt-long sequence (GGG GAA​GCG​GGC​ATG​CGG​CCA​GCC​ATA​GCC​GAT​CA).

Typical *in vitro* transcription reaction (80 µl) was incubated at 37°C for 4 h and contained RNA polymerase buffer (40 mM Tris HCl pH 7.9, 10 mM MgCl_2_, 1 mM DTT, 2 mM spermidine), 10 U/µL T7 polymerase (ThermoFisher Scientific, HC, 200 U/µl), 1 U/µl RiboLock RNase inhibitor (ThermoFisher Scientific), 0.5 mM CTP/UTP/GTP or CTP/UTP/ATP with 0.125 mM ATP or GTP for 0.1 µM A φ 2.5 or G φ 2.5 DNA template respectively—and 0.375–1.125 mM cap analog of interest (3- to 9-fold excess). Following 4 h incubation, the template was removed by treatment with 1 U/µL DNase I for 30 min at 37 C. The obtained RNAs were purified using RNA Clean & Concentrator-5 (Zymo Research). Transcript homogeneity was analyzed on 15% acrylamide/7 M urea gels/1⨯ TBE and stained with SYBR Gold, whereas the concentration was determined spectrophotometrically.

#### 2.3.2 DNAzyme cleavage and capping efficiency determination

To generate homogenous 3′-ends, RNAs were first subjected to HPLC purification using Clarity® 3 µM Oligo-RP phenomenex column at 50 °C, 1 ml/min on Shimadzu apparatus. A linear gradient from 10% to 30% of 200 mM TEAAc pH7/ACN 1/1 in 100 mM TEAAc pH 7.0 was applied. The collection of RNA samples was performed in a way that both capped and uncapped RNA fractions were pooled together (uncapped and capped RNA species co-eluted in all cases, except the FAD derivatives). The collected RNAs were freeze dried twice before the DNAzyme treatment. For that purpose, the HPLC purified 35-nt-long transcripts (1 µM) were incubated with 1 µM DNAzyme 10-23 [TGA​TCG​GCT​AGG​CTA​GCT​ACA​ACG​AGG​CT-GGC​CGC, (Schubert et al., 2003; Coleman et al., 2004)] in 50 mM MgCl_2_ and 50 mM Tris-HCl pH 8.0 for 2 min at 95°C followed by 1 h at 37°C, then DNase I treatment was performed, which allowed to produce 3′-end homogenous 25 nt RNAs. The homogeneity and concentration of the prepared RNAs were analyzed as above. Additionally the transcripts were analyzed on a 1% acryloylphenyl boronic acid in 15% acrylamide/7 M urea gel/1⨯ TBE, with SYBR Gold staining (Nubel et al., 2017). The capping efficiency values were determined based on densitometric quantification of the major bands intensity (*I*_*capped*_ and *I*_*uncapped*_) corresponding to capped and uncapped RNAs according to the equation:

$$Capping\;efficiency\;=\frac{I_{capped}\;}{I_{capped}+I_{uncapped}}100\%$$

#### 2.3.3 FAD-CapQ assay

FAD-CapQ analysis for capping efficiency of FAD-capped RNAs was performed as previously reported. (Doamekpor et al., 2020) Briefly, 3 µg of RNA was digested SpRai1 (200 ng) in 15 μl of reaction buffer containing 50 mM Tris (pH 7.9), 100 mM NaCl, 10 mM MgCl_2_ and 1 mM DTT at 37 °C for 2 h to release intact FAD. FAD standard curve was prepared under the same reaction conditions using increasing amount of FAD-capped RNA obtained in the presence of 9 equiv. of FADpG. To quantify the released FAD, an FAD assay kit (FAD Colorimetric/Fluorometric Assay Kit, BioVision) was used according to the manufacture’s protocol.

#### 2.3.4 HPLC-MS qualitative and semi-quantitative analysis

##### 2.3.4.1 RNA preparation

Typical *in vitro* transcription reaction (20 µL) was incubated at 37°C for 3 h and contained RNA polymerase buffer (40 mM Tris HCl pH 7.9, 10 mM MgCl_2_, 1 mM DTT, 2 mM spermidine), 10 U/µl T7 polymerase (ThermoFisher Scientific, HC, 200 U/µl), 0.0125 U/µl pyrophosphatase (ThermoFisher Scientific), additional 15 mM MgCl_2_, 1 U/µl RiboLock RNase inhibitor (ThermoFisher Scientific), and either 1) 5 mM CTP/UTP/GTP and 4 mM ATP and 10 mM cap analog of interest (2.5-fold excess of FAD or FADpG) for 40 ng/µl A φ 2.5 DNA template or 2) 5 mM CTP/UTP/ATP with 4 mM GTP and 10 mM cap analog (GlcppUpG or NAcGlcppUpG) for 40 ng/µl of G φ 6.5 DNA template. Following 3 h incubation, the template was removed by treatment with 1 U/µl DNase I for 30 min at 37°C. The obtained RNAs were purified using 500 µg Monarch RNA clean-up kit (New England Biolabs). Transcript homogeneity was analyzed on 15% acrylamide/7 M urea gels/1⨯ TBE and stained with SYBR Gold, whereas the concentration was determined spectrophotometrically.

##### 2.3.4.2 Preparation of the chromatographic system

Before analyses and between each series of three technical repetitions the chromatographic system required a washing with 1% phosphoric acid (v/v) in 50% aqueous methanol for 2 h and further 50% aqueous methanol for another 2 h to remove all traces of metal cations unspecifically bound within the system (Birdsall et al., 2016).

##### 2.3.4.3 General settings

All analyses were performed on Agilent system (1,260 series) equipped with diode array detector (recording at 260 nm) and mass spectrometer (AB Sciex QTRAP3200) in reversed phase mode (Waters ACQUITY UPLC BEH C18 Column, 130 Å, 1.7 µm, 2.1 mm × 100 mm). Analytical method was elaborated on the basis of previously published conditions (Levin et al., 2011). Flow rate was fixed at 0.2 ml/min and a linear gradient of 0–40.6% of phase B in 70 min was pumped through the thermostated column at 55°C. Composition of mobile phases were as follows: phase A—5 mM TEA, 5 mM PA (propylamine) in 10% aqueous methanol with addition of 25 ml of HFIP (1,1,1,3,3,3-Hexafluoropropan-2-ol) per 0.5 L of buffer, pH 7.5; phase B—5 mM TEA, 5 mM PA in 40% aqueous methanol with addition of 25 ml of HFIP per 0.5 L of buffer, pH 8.0. Mass spectrometer settings were: curtain gas: 10 psi, temperature: 350°C, gas1: 60 psi, gas2: 50 psi. Ions were scanned through linear ion trap (range 600-1,300 Da) in Enhanced Multi-Charged mode: fill time 40 msec, Q3 Entry Barrier 5 V, Empty Time 4 msec, MSC Barrier 5 V.

A portion of 10 μl of RNA solution (conc. c.a. 300 ng/μl, determined spectrophotometrically, ε_260_ = 0.025 (μg/ml)cm^−1^) was injected per single analysis.

##### 2.3.4.4 Data processing

For qualitative analyses mMass open source program was used (http://www.mmass.org/, (Strohalm et al., 2010)) in order to deconvolute spectrograms (Supplementary Figures S1–S4). ChemCalc open source protocol was used for theoretical molecular weight calculation (https://www.chemcalc.org/, (Patiny and Borel, 2013)). All signals were assigned to a general pseudomolecular ion formula [M-xH + yNa]^(x+y)^: for deconvoluted data [M-15H + 14Na]^-1^; for signals on spectrogram [M-24H + 14Na]^-10^, [M-25H + 14Na]^-11^, [M-26H + 14Na]^-12^, etc. Qualitative analyses were based on comparison of theoretical data and experimental with accuracy of few daltons (mediane 50 ppm—Supplementary Table S1) for deconvoluted mass spectrum. Data was listed in Supplementary Table S1.

For semi-quantitative analyses Analyst 1.6.3 was used. The most intense signals on the spectrogram of each RNA type were integrated with IntelliQuant mode (peak split factor 5 or 10, minimum height 5.0e5).

Capping efficiency was determined based on the ratio of area corresponding to total capped RNAs versus total capped and uncapped RNAs, which have been identified by means of qualitative analyses. The mean value was calculated from three technical replicates.

## 3 Results

### 3.1 Chemical synthesis of non-canonical capping reagents

**FIGURE 1:**
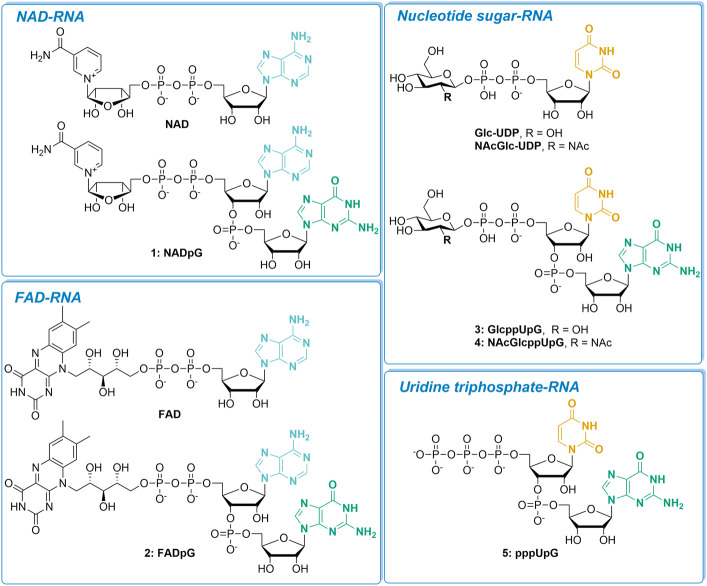
The set of nucleotide derivatives used for *in vitro* transcription studies, divided into four categories according to the type of RNA 5′-end they produce.

We evaluated a set of five transcription initiating nucleotides (NADpG, FADpG, GlcppUpG, NAcGlcppUpG, and pppUpG; compounds **1–5**, respectively) and studied their incorporation into RNA in comparison to their unmodified counterparts, NAD, FAD, GlcUDP and NAcGlcUDP (Figure 1). We divided these nucleotides into four groups based on the type of 5′-capped RNA they produce: NAD-RNA, FAD-RNA, nucleotide sugar-RNA, or triphosphate-U-RNA.

**SCHEME 1:**
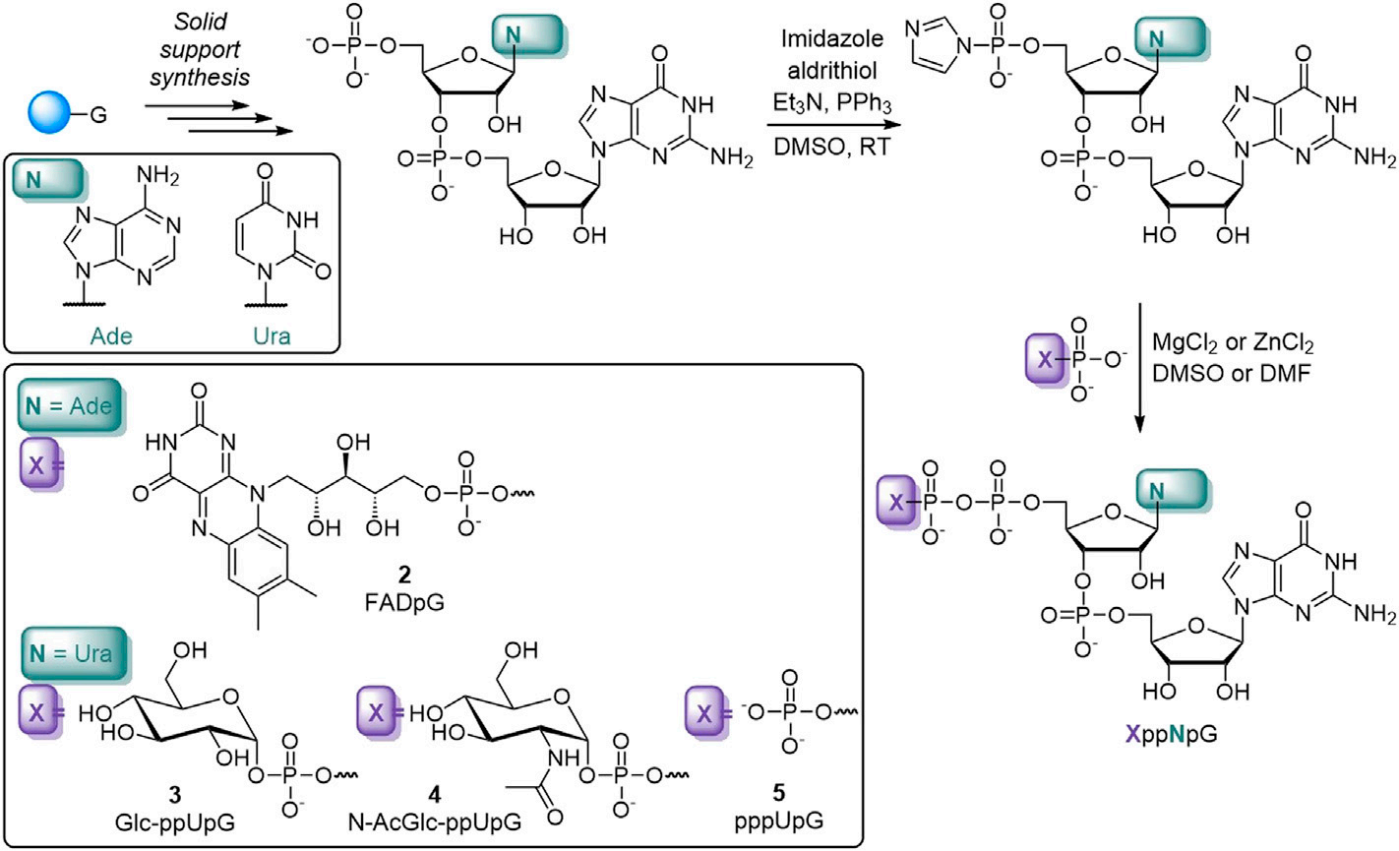
Representative syntheses of the non-canonical caps used in this study.

To obtain compounds **1–5**, we employed a synthetic strategy relying on the coupling reaction between a 5′-phosphorimidazolide derivative of mono- or dinucleotide and the appropriate phosphate nucleophile (Scheme 1) (Jemielity et al., 2003; Dabrowski-Tumanski et al., 2013; Mlynarska-Cieslak et al., 2018). In brief, solid-supported synthesis using phosphoramidite chemistry was performed to obtain the appropriate dinucleotide 5′-monophosphates (61–64% yield). The latter were then converted into 5′-monophosphorimidazolides, which were isolated as sodium salts by precipitation from acetone (almost quantitative). Finally, the appropriate imidazole-activated phosphate derivative was reacted with a phosphate nucleophile in the presence of divalent metal chloride to produce the corresponding final derivative, purified first by Sephadex and then by preparative HPLC (18–80% yield). For the majority of the compounds, ZnCl_2_ was the most efficient mediator of pyrophosphate bond formation, with the exception of nucleotide sugar derivatives, which were unstable in the presence of ZnCl_2_ but were efficiently formed in the presence of MgCl_2_ (Dabrowski-Tumanski et al., 2013).

### 3.2 Co-transcriptional capping strategy

**FIGURE 2:**
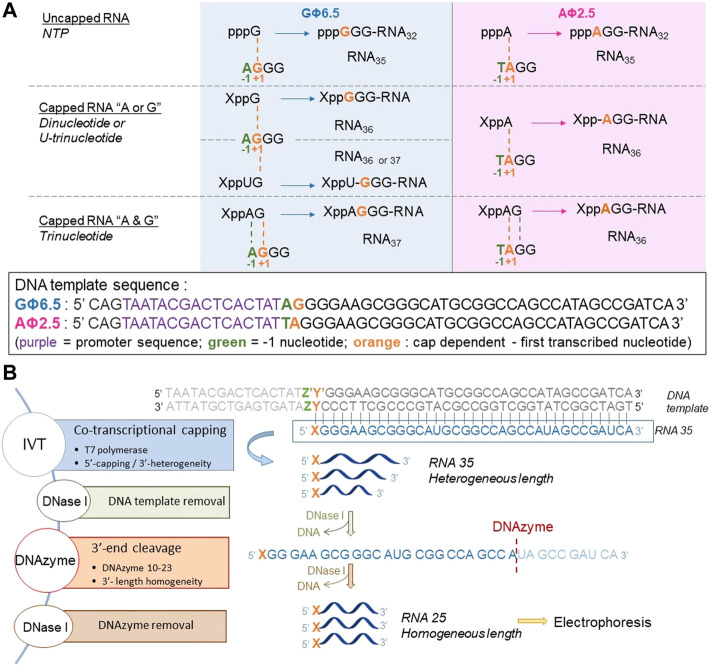
**(A)** Transcription initiation events expected for the φ 2.5 and φ 6.5 T7 promoters and different initiating nucleotides; For clarity, only the coding (sense) strands of the promoter regions of double-stranded DNA templates are shown. **(B)** General *in vitro* transcription protocol for the assessment of nucleotide analog incorporation.

To assess the incorporation of different analogs into the RNA 5′ end, we analyzed the products of *in vitro* transcription reactions in the presence of various concentrations of the initiating nucleotide from templates containing the φ 2.5 (...T^-1^A^+1^G^+2^G^+3^) and φ 6.5 (...A^-1^G^+1^G^+2^G^+3^) promoters (Figure 2A). The dinucleotides containing only A (marked in blue in Figure 1; NAD and FAD) were expected to be incorporated into RNA only during transcription from φ 2.5 promoter (where TSS or +1 position is A), producing a mixture of 5′ capped and uncapped RNAs, as reported previously (Huang, 2003). The corresponding trinucleotides (NADpG and FADpG respectively), composed of both adenosine (blue) and guanine (green) moieties, were expected to be incorporated into RNA during transcription from both φ 2.5 and φ 6.5 promoters owing to the double pairing of A and G moieties with +1/+2 and -1/+1 positions in these promoters, respectively (Figure 2A), whereby the RNAs produced during transcription from the φ 6.5 promoter are 1 nt longer than those obtained by transcription from φ 2.5. Nucleotide sugars containing uridine (U, marked in orange in Figure 1; GlcUDP and NAcGlc-UDP) cannot be incorporated into RNA by transcription from either the φ 2.5 or G φ 6.5 promoters because of the lack of complementarity with the DNA sequence at or near the TSS. In contrast, the uridine and guanine-containing derivatives (G marked in green in Figure 1; GlcppUpG, pppUpG, and NAcGlcppUpG) were expected to act as transcription initiators in the presence of the φ 6.5 promoter, owing to the complementarity of guanine with the TSS.

**FIGURE 3:**
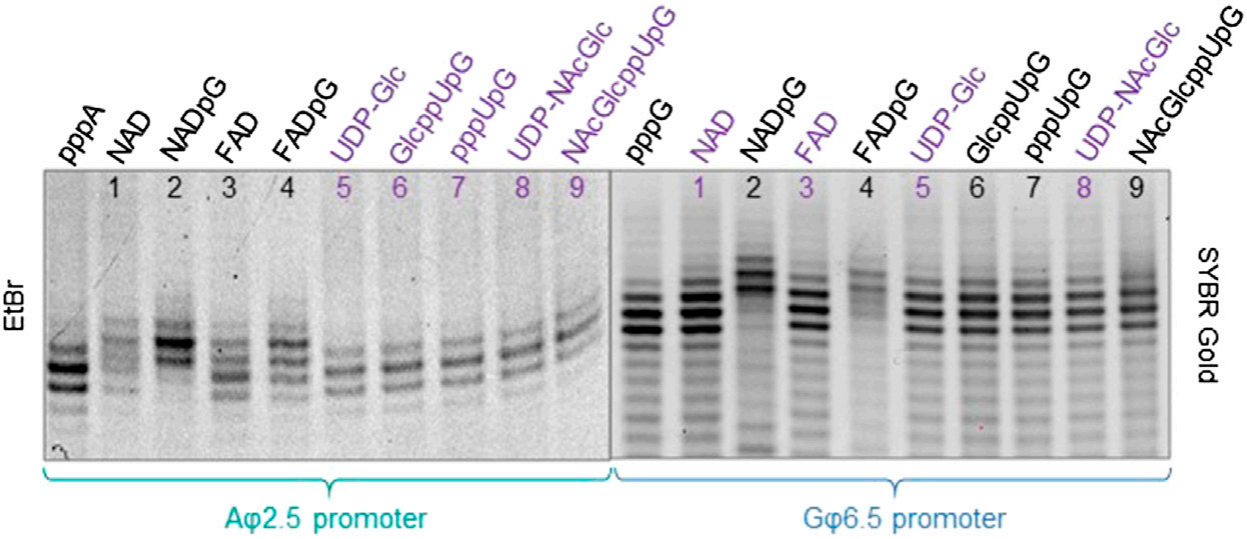
High resolution polyacrylamide gel electrophoresis (HRPAGE; 15% PAA, 7 M urea, 1⨯ TBE) analysis of 35 nt IVT RNAs obtained from templates containing either the A φ 2.5 promoter (left gel stained with Ethidium Bromide—EtBr) or the G φ 6.5 promoter (right gel stained with SYBR Gold) in the presence of 0.5 mM NTPs, 0.125 ATP/GTP, and 0.375 mM of the initiating nucleotide analog. The purple labels correspond to the caps that are expected not to incorporate into RNA with the defined promoter.

To confirm that the synthesized initiating nucleotides are incorporated in the anticipated promoter-specific manner, we performed pilot transcription reactions from two DNA templates, encoding a 35-nt long RNA and differing only in the promoter sequence (φ 2.5 or φ 6.5). To ensure more efficient capping with the initiating nucleotides, the concentration of NTP corresponding to the TSS in the promoter sequence (i.e., ATP for φ 2.5 and GTP for φ 6.5 promoter) was lower than that of other NTPs (0.125 *versus* 0.5 mM), while the concentration of the initiating non-canonical cap analog was elevated (3-fold relative to the initiating NTP). The resulting RNA was analyzed by denaturing polyacrylamide gel electrophoresis (PAGE; Figure 3). The gels were stained differently, revealing SYBR Gold as a better choice for gel quality than EtBr, therefore used for the following PAGE staining. Transcripts were, as expected, significantly heterogeneous (Pleiss et al., 1998; Coleman et al., 2004), even in the case of uncapped RNA, which significantly impaired quantitative analysis. In addition to 35-nt long RNA, at least two more bands were observed, corresponding to 34- and 36-nt long RNA, below and above the 35-nt long RNA, respectively. Worth noticing, the heterogeneity of the sample was higher when using φ 6.5 rather than φ 2.5 promoter, in agreement with earlier studies (Coleman et al., 2004). However, the migration of non-canonical capped RNA, such as analyzed in this study, is dependent not only on RNA “size” but also on the physicochemical properties of the cap, and hence, is to some extent unpredictable. The positive charge of nicotinamide, the linear sugar in flavin derivatives, or the presence of glucose moiety differ from standard ribonucleotides and may have different impact on the RNA mobility in the gel than canonical nucleotides. As a consequence, we observed significantly higher-migrating bands for FAD/NAD derivatives, while no noticeable capped bands for glucose derivatives. Nevertheless, it was possible to confirm the incorporation of NADpG and FADpG (Figure 3, lanes 2 and 4 respectively) into RNA from templates containing both the φ 2.5 and φ 6.5 promoters. Unfortunately, it was difficult to assess the incorporation of all uridine and adenine analogues because of the low difference in mobility between capped and uncapped RNA species and the high sample heterogeneity, respectively. In order to reduce the 3’ heterogeneity of the sample, DNAzymes or ribozymes have been widely employed (Schurer et al., 2002), where DNAzyme was further used in our work.

### 3.3 Capping efficiency using G (φ 6.5) promoter

**FIGURE 4:**
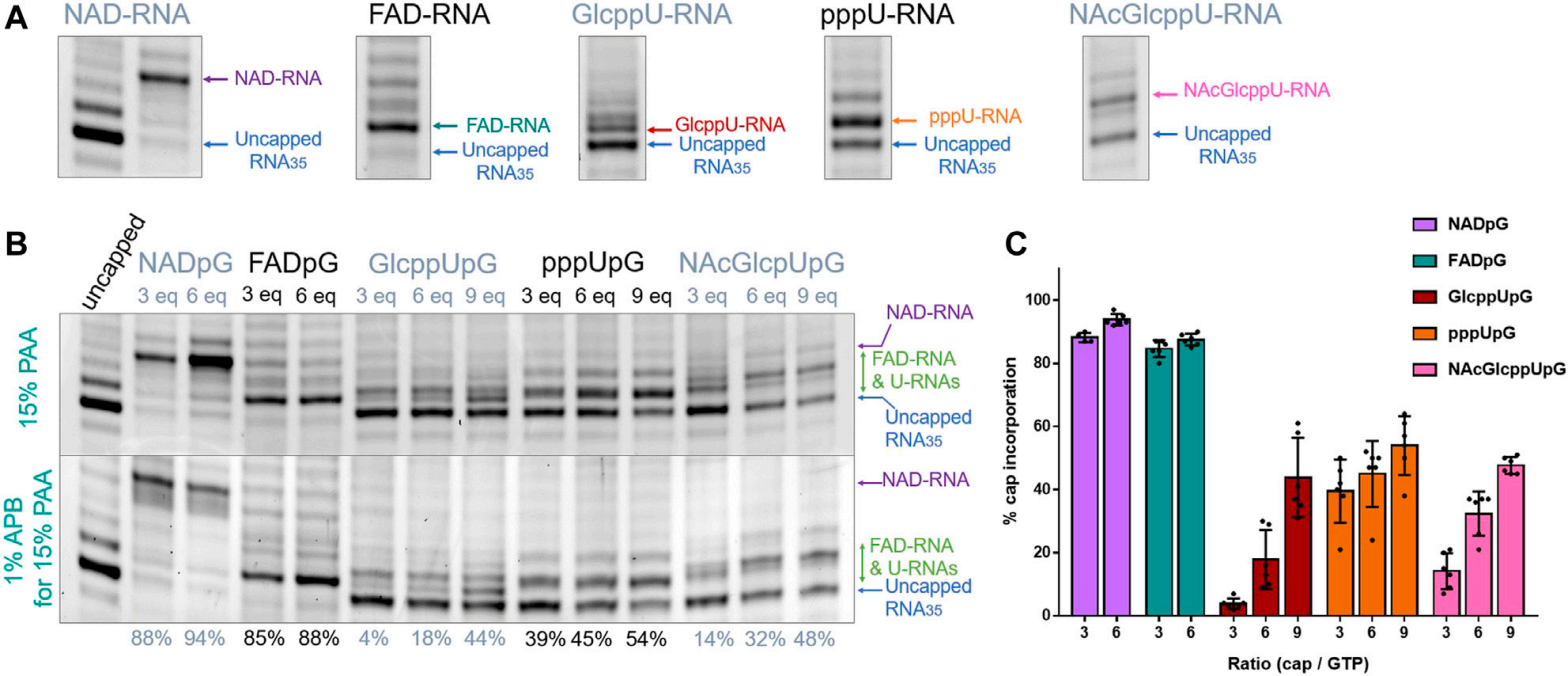
**(A)** HRPAGE (15% PAA, 7 M urea, 1⨯ TBE, SYBR Gold) analysis of differently capped RNA species with indication of bands used for capping efficiency assessment. **(B)** Representative HRPAGE (15% PAA, 7 M urea, 1⨯ TBE, SYBR Gold), without APB (upper gel) or with 1% APB (bottom gel), of 25 nt IVT RNA obtained from a template with G φ 6.5 promoter after 3′ end trimming steps. The capping efficiency values given below the gel indicate the mean values from triplicate analyses performed on three independent transcriptions and 6 densitometric measurements (+/- APB). **(C)** Comparison of the capping efficiencies under different conditions as a function of [nucleotide analog]/[GTP] ratio.

Since the heterogeneity of RNA samples obtained with polymerase T7 arises mostly from the 3′ ends (Helm et al., 1999), to enable quantitative assessment of capping efficiencies, we trimmed the 3′ ends of crude RNAs using DNAzyme 10-23 (Figure 2B) (Coleman et al., 2004) on HPLC purified RNAs. This purification was necessary in order to provide high efficiency DNAzyme cleavage. It has to be noted that we used a previously described HPLC purification method to purify our IVT RNAs (Depaix et al., 2021), eliminating impurities from IVT, where most capped and uncapped transcripts were co-eluting, except for FAD-RNAs. Assessing the capping efficiency required keeping uncapped and capped RNAs combined for further analysis, so the HPLC conditions were not optimized further. The high resolution (HR) polyacrylamide gel electrophoresis (PAGE) analysis of the trimmed products enabled sufficient separation of capped and uncapped RNA species in most cases. Using this improved protocol, we first assessed the incorporation of all guanine-containing trinucleotides **1–5** using a DNA template with the φ 6.5 promoter. The transcription was performed in the presence of different concentrations of the initiating nucleotide analog (0.375, 0.75, and optionally 1.125 mM, i.e., 3-, 6-, and 9-fold excess of GTP, respectively), followed by 3′ end trimming. The resulting 25 nt RNAs were analyzed using HRPAGE (Figure 4). Unfortunately, even though the 3′ heterogeneity was removed, the 5’ heterogeneity remained and several RNA bands were still visible in the gel. Combined with the unexpected migration of capped-RNAs, the assessment of capping efficiency was not as straightforward as we expected. Hence, we based our calculations on comparing the most intense uncapped RNA band (the migration level of which is known thanks to the uncapped 25 nt long RNA reference), and the most intense capped band (not present in the uncapped sample), as depicted in Figure 4A showing 15% PAA gel analysis of capped and uncapped RNAs. NADpG and FADpG at 0.75 mM concentration were incorporated into RNA with very high efficiency (capping efficiencies determined by densitometry of 94 and 88%, respectively), whereas uridine derivatives (GlcppUpG, pppUpG, and NAcGlcppUpG) had lower incorporation efficiency (18, 45, 32%, respectively). We, therefore tested the higher excess of the uridine analogs **3**, **4** and **5** (1.125 mM, 9-fold excess), which yielded incorporation efficiencies close to 50% (44%, 48%,, and 54% respectively).

In addition to standard electrophoresis, we have also analyzed the samples in polyacrylamide gels containing 1% acryloyl phenyl boronic acid (APB). ABP has been used to differentiate the mobility of capped and uncapped RNAs due to interactions with *cis*-diol groups, which are present at the RNA 3′-ends and optionally at the 5′-ends if the RNA is 5′ capped (Nubel et al., 2017; Sato et al., 2018). Hence, slower migration through the APB gels is expected for capped RNAs (due to one more *cis*-diol function within the RNA) compared to uncapped RNA, allowing an easier separation and assessment of incorporation. Interestingly, analysis of differently modified RNAs in APB gels revealed that a significant improvement in the separation between capped and uncapped RNA was observed only for NAD derivatives, whereas for FAD and nucleotide sugar derivatives, the effect of APB was barely notable (Figure 4B). We hypothesize that this is because the sugar moiety in FAD is acyclic and therefore much more flexible than the ribose moiety in NAD, while the glucopyranose in UDP-Glc contains only *trans*-diol moieties, indicating that the proper spatial arrangement and conformational rigidity of the diol is a prerequisite for efficient interaction with APB.

### 3.4 Capping efficiency using A (φ 2.5) promoter

**FIGURE 5:**
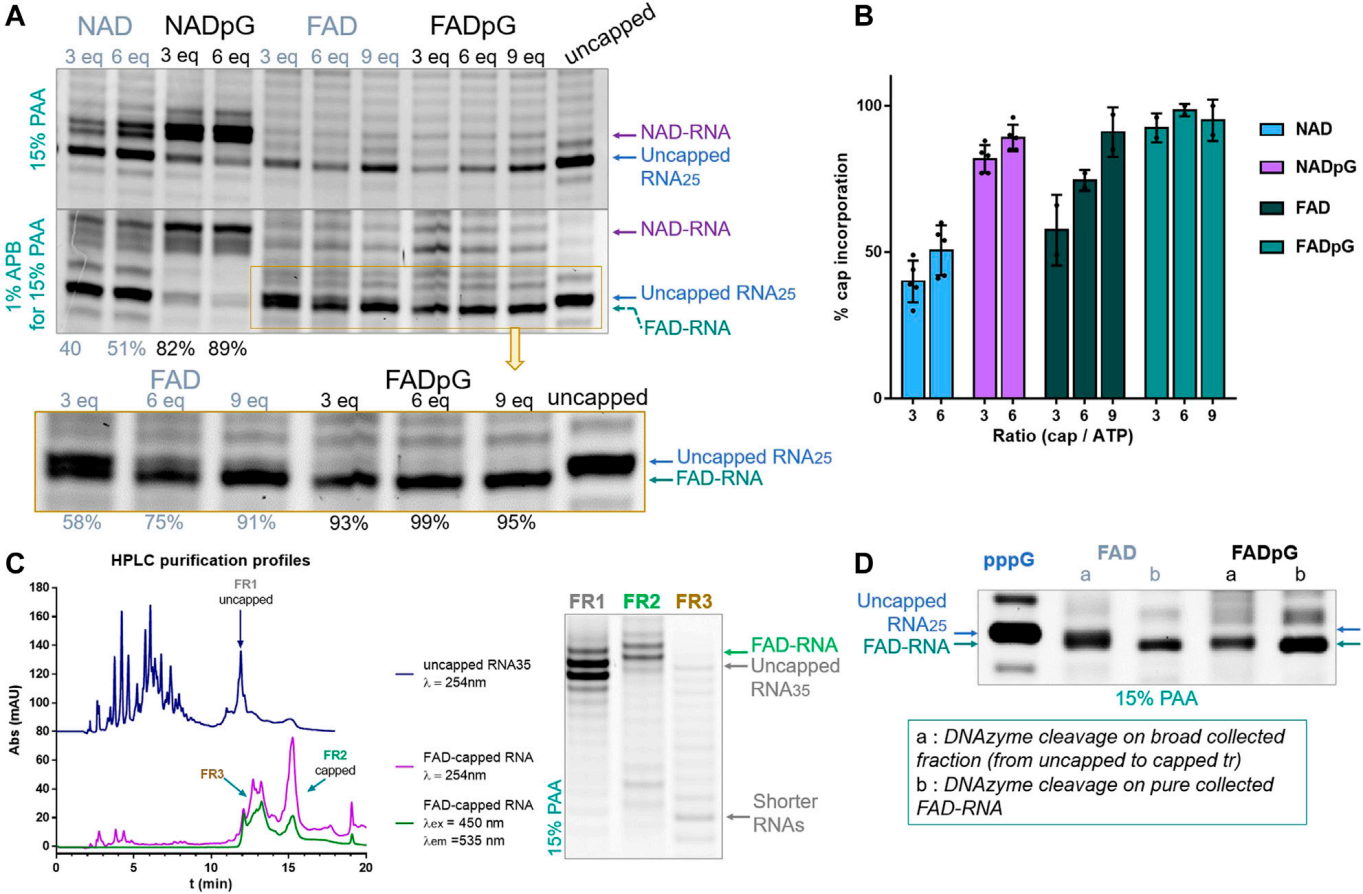
**(A)** Representative HRPAGE (15% PAA, 7 M urea, 1⨯ TBE, SYBR Gold), without APB (upper gel) or with 1% APB (bottom gel), of 25 nt IVT RNA obtained from a template with the φ 2.5 promotor after 3′ end trimming steps. The capping efficiency values given below the gel indicate the mean values from triplicate analyses performed on three independent transcriptions and 6 densitometric measurements (+/- APB) for NAD and NADpG, and from duplicate analyses FAD and FADpG (only APB). **(B)** Comparison of capping efficiencies under different conditions as a function of nucleotide analog / GTP ratio. **(C)** HPLC purification profiles of IVT uncapped and FAD-capped RNAs, with corresponding HRPAGE analysis (15% PAA, 7 M urea, 1⨯ TBE, SYBR Gold) of the collected fractions. **(D)** HRPAGE (15% PAA, 7 M urea, 1⨯ TBE, SYBR Gold) of RNAs after DNAzyme trimming on uncapped, FAD initiated and FADpG initiated IVT RNAs.

Similarly, we tested the incorporation of adenine-containing nucleotides, namely NAD, NADpG, FAD, and FADpG, using 3-, 6-, or 9-fold excess over ATP during transcription from a template containing the φ 2.5 promoter (Figure 5). Again, the 3′-trimmed RNAs were analyzed by standard 15% PAA electrophoresis, and with an additional 1% APB (Figure 5B). As expected, we found that NADpG was incorporated more efficiently into the 5′ end of RNA than NAD. Surprisingly, PAGE analysis of RNA samples prepared in the presence of FAD and FADpG suggested these analogs were not incorporated (absence of capped band on the gel; Figure 5A), even if a 9-fold excess of these derivatives was applied. However, when the same samples were analyzed in APB gels, we noticed in some cases two closely migrating RNA bands. Based on the changing band intensities as a function of FAD analog, we concluded that FAD and FADpG were incorporated into RNA, but surprisingly, FAD-RNA migrated at the same level as uncapped RNA during standard PAGE, while migrating slightly faster than uncapped RNA in APB gels (Figure 5A, yellow inset). The result did not change significantly when we tested different polyacrylamide and APB concentrations, even at the conditions which previously enabled separation of FAD-capped and decapped (i.e., 5′-monophophorylated) RNAs. (Doamekpor et al., 2020) This unexpected migration of 25 nt FAD-capped RNA was confirmed by 3′-end trimming of HPLC-purified 35 nt FAD-capped RNA (Figure 5C). Indeed, the HPLC purification profile of uncapped RNA (dark blue line, Figure 5C) showed that the retention time (tr) of uncapped-RNA_35_ was around 11.9 min (FR 1, Figure 5C) whereas the HPLC purification profile of FAD-capped RNA showed a main peak round 15.4 min (FR 2) and another wide peak around 12-14 min (FR 3). This HPLC profile (FAD-RNA, Figure 5C) corresponds to the IVT sample prepared with 9-fold excess of FADpG compared to GTP. Though the UV-signal (λ = 254 nm; Figure 5C, purple line) of FR 3 was close to the retention time of uncapped RNA (tr = 11.9 min, FR1), the signal was also detected by the fluorescence detector set up for FAD fluorescence (λex = 450 nm, λem = 535 nm, green line, Figure 5C). Hence, we assumed that this peak (FR 3) contained mainly shorter FAD-RNA species and that the amount of uncapped RNA, if there was, was rather negligible. Further HRPAGE of the 3 distinct fractions confirmed the presence of uncapped and capped RNAs for FR1 and FR2, respectively, and confirmed that FR3 contained only traces of uncapped RNA with major products of shorter lengths. We further compared the DNAzyme trimming on two different FAD-RNA samples, one from the use of 6-fold excess of FAD and the second when capping with 9-fold excess of FADpG. The DNAzyme cleavage was performed either on the combined fractions from HPLC (lanes a, Figure 5D, combined uncapped and capped RNAs) or on pure FAD-RNA (lanes b Figure 3D). Pure FAD-RNAs, after 3′-cleavage, provided a single main band corresponding to the FAD-capped RNA, confirming a slightly faster migration than the uncapped 25 nt long RNA. Moreover, we could clearly observe a thicker band of RNA obtained from the capping with FAD than with FADpG (FAD lane a, compared to FADpG lane a, respectively, Figure 5D), due to higher amount of uncapped RNA in the first sample. This is in agreement with our observation that dinucleotide cap incorporation was less efficient than the corresponding trinucleotide incorporation. Although we were able to assess FAD and FADpG capping efficiencies based on the intensities of the two bands determined by APB-PAGE, this unusual migration behavior prompted us to confirm our findings using an alternative methods.

**FIGURE 6:**
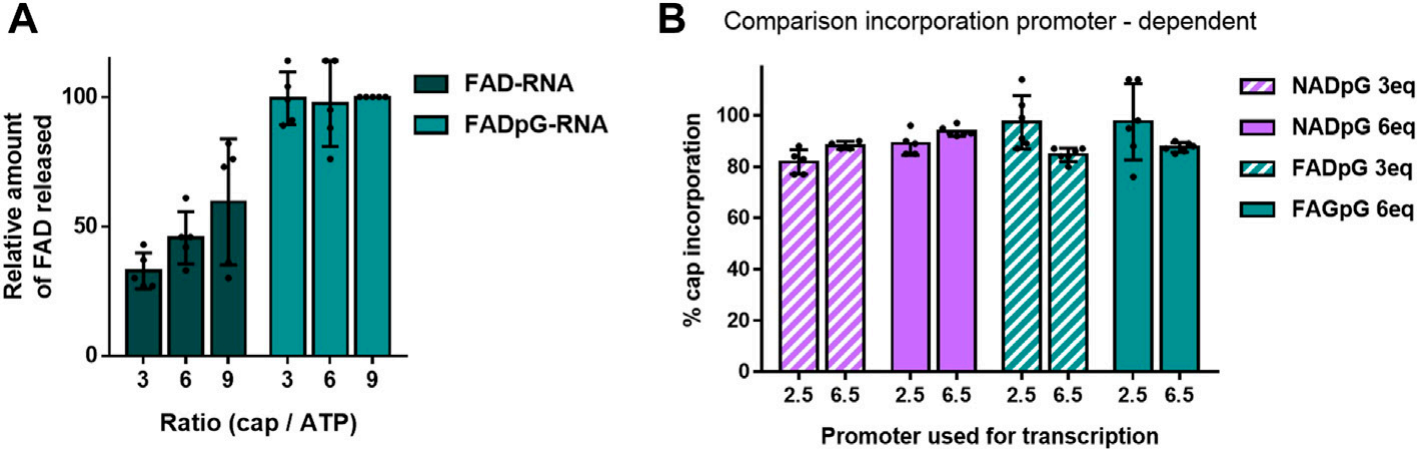
**(A)** Capping efficiencies of FAD- and FADpG-RNAs determined using the FAD-capQ method (using 9x excess FADpG). **(B)** Capping efficiencies obtained for NADpG and FADpG (both quantification methods included) as a function of the T7 promoter.

The FAD-capQ assay (Doamekpor et al., 2020) was used to quantify FAD in each of the six RNA types (FAD-RNA/FADpG-RNA 3/6/9eq). FAD-capped 35-nt RNAs were first subjected to SpRai1 enzyme digestion to release intact FAD. Then, a fluorometric measurement of the samples after overnight incubation with FAD Assay Buffer (FAD Colorimetric/Fluorometric Assay Kit, BioVision), revealed fluorescence signal proportional to the concentration of FAD. For these experiments, the RNA sample obtained with a 9-fold excess of FADpG was set to 100%, which was close to the actual capping efficiency value according to our findings from HPLC and HRPAGE (Figures 5C,D). This sample was then used as a reference to calculate the relative FAD contents in other samples (Figure 6A).

The FAD-capQ assay demonstrated the presence of FAD in the samples, thereby confirming their presence at the 5′-end of RNAs. Moreover, the relative capping efficiency values determined by electrophoresis and FAD-capQ were in a very good agreement, supporting the interpretation of the unusual migration of FAD-RNA in the gels. Moreover, the efficiency of incorporation of FAD increased in conjunction with the FAD/ATP ratio, which is consistent with what was expected based on the results for NAD, whereas FADpG provided significantly higher capping even when used at a 3-fold excess of ATP. Furthermore, when comparing the incorporation of the two adenine-containing trinucleotides—NADpG and FADpG—with both promoters A (φ 2.5) and G (φ 6.5), we observed a similar incorporation efficiency but slightly better incorporation of NADpG when the G (φ 6.5) promoter was used, in contrast to FADpG, which showed a better efficiency using the A (φ 2.5) promoter (Figure 6B).

**FIGURE 7:**
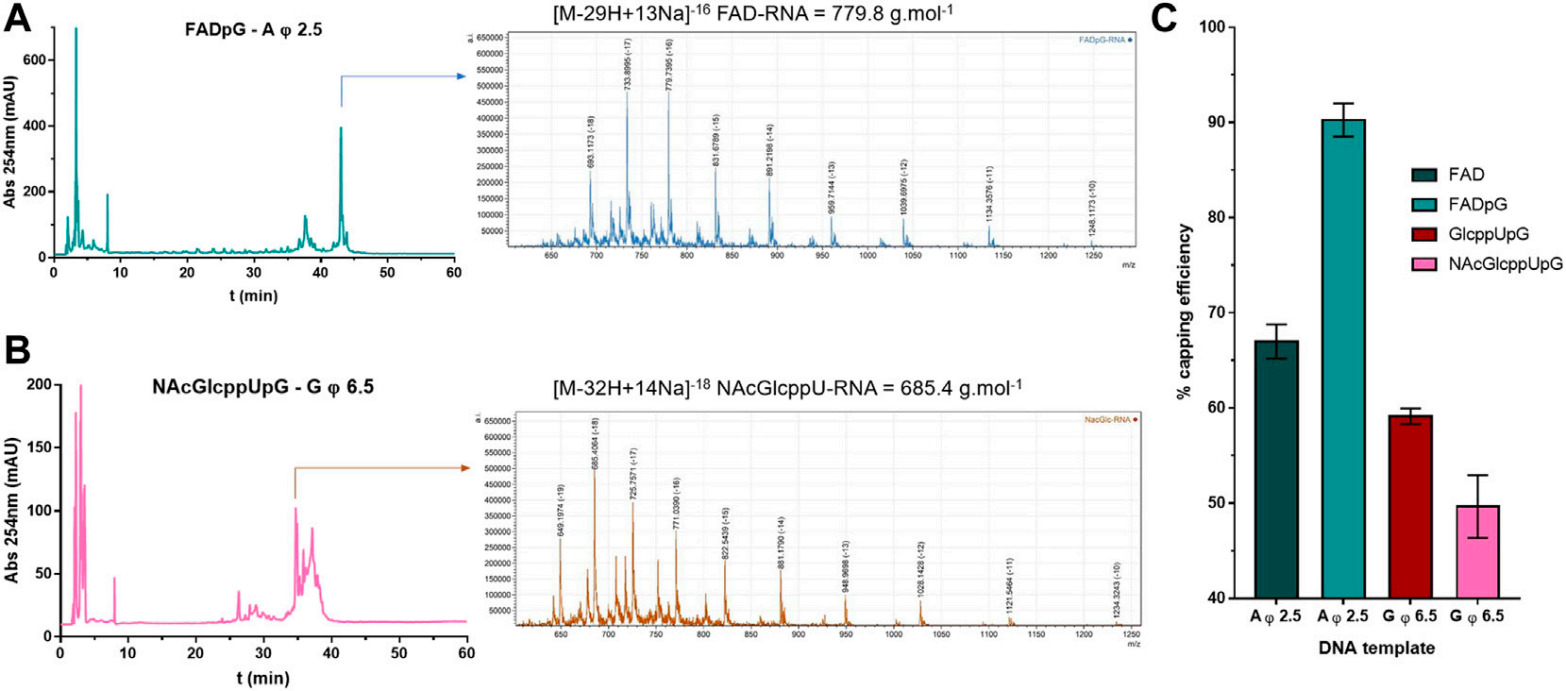
**(A)** HPLC chromatogram and MS spectrogram issued from LC-MS analyses on FADpG initiated IVT RNA using A φ 2.5 DNA template. **(B)** HPLC chromatogram and MS spectrogram issued from LC-MS analyses on NAcGlcppUpG initiated IVT RNA using G φ 6.5 DNA template. **(C)** Capping efficiencies for FAD, FADpG, GlcppUpG and NAcGlcppUpG calculated from LC-MS analyses, as mean values from 3 technical repetitions.

As a final verification of the capping efficiencies for our novel RNA 5’ end analogs, we performed an LC-MS analysis of 35 nt FAD-RNAs, GlcppU-RNAs, and NAcGlcppU-RNAs obtained by IVT under promoters specified in Figure 6. To obtain sufficient amount of material for the analyses we developed a modified IVT protocol employing higher nucleotide concentrations and 2.5-fold excess of the cap analog over the standard initiating NTP (for details see Materials and Methods). The LC-MS analyses enabled us to identify all distinct capped- and uncapped RNA species (Supplementary Table S1, Supplementary Figures S1–S4) and determine overall capping efficiency based on the ratio of total capped versus total RNA (Figure 7, Supplementary Figure S5). Figures 7A,B shows a representative HPLC chromatograms and the corresponding MS spectrograms for RNA capped with FADpG (obtained with A φ 2.5 promoter) and NAcGlcppUpG-capped RNA (obtained with G φ 6.5), respectively. Although the RNAs were obtained using a different protocol than RNAs analyzed above, the obtained results were in very good qualitative agreement with results from HRPAGE analyses. The highest capping efficiency was found for FADpG (90%), whereas FAD under similar conditions yielded only 67% capping. GlcppUpG and NAcGlcppUpG afforded capping efficiencies of 59 and 49%, respectively (Figure 7C), confirming that these analogs afford slightly decreased capping efficiency due to imperfect pairing with the promoter sequence.

## 4 Discussion

Herein, we designed and synthesized a set of di- and trinucleotide derivatives of various non-canonical RNA 5′-ends straightforwardly and efficiently. In cases where dinucleotides could not be incorporated into RNA with the usual polymerase/promoter couple, the corresponding trinucleotides were useful and showed good to very good incorporation into RNA by T7 polymerase (Table 1). For instance, NADpG and FADpG displayed notably higher incorporation (close to 95%) comparing to NAD and FAD, respectively, and acted as transcription initiators from both the φ 2.5 and φ 6.5 promoters, whereas NAD and FAD were only moderate substrates for T7 polymerase using the A φ 2.5 promoter. Moreover, none of the uridine-containing derivatives were incorporated into RNA, but their corresponding uridine-guanine counterparts were incorporated with approximately 50% yield under optimized conditions using the G φ 6.5 promoter. We further identified problems that can occur during the electrophoretic analysis of non-canonically capped RNAs, especially FAD-RNAs, highlighting the need for cautious consideration of the results. The relatively low capping efficiencies achieved for uridine derivatives resulting in significant amount of uncapped RNA in the samples may limit some of the potential applications or necessitate further work-up of the samples. For short transcripts, this could be potentially achieved by the use of RP or ion exchange HPLC chromatography under conditions resolving uncapped and capped transcripts. Otherwise, post-transcriptional enzymatic processing such as a successive polyphosphatase and 5′-3′-exonucleoase treatments, should allow to degrade the 5′-triphosphate RNA, keeping the 5′capped RNA intact (Sikorski et al., 2020). Overall, the use of trinucleotides provides several noticeable advantages for *in vitro* transcription, thus promoting the prospect of more efficiently introducing various non-canonical RNA 5′-caps and providing access to molecular tools that could help understand the biological functions of non-canonically capped RNAs. For instance, 3′ biotinylation of non-canonically capped RNAs could yield probes for pull-down assays, to provide information on proteins interacting with the corresponding 5′ caps. Future research may also involve the application of this methodology for non-canonically capped mRNA synthesis in order to assess its biological functions and translational properties. Further studies on longer RNA templates and novel analytical methods would also be useful in the future to determine the full scope and limitations of this capping technology.

**TABLE 1:** Summary of RNA capping efficiencies for the initiation of different nucleotides as a function of the T7 promoter present in the DNA template.

Compound	RNA 5′ end type	Promoter compatibility		Optimal excess over initiating nucleotide		Capping efficiency	
		φ 6.5^a^	φ 2.5^b^	φ 6.5^a^	φ 2.5^b^	φ 6.5^a^	φ 2.5^b^
		(GGG)	(AGG)	(GGG)	(AGG)	(GGG)	(AGG)
NAD	NAD-RNA	no	yes	-	**6 equiv**	0	**51%**
NADpG		yes	yes	**6 equiv**	6 equiv	**94%**	89%
FAD	FAD-RNA	no	yes	-	**9 equiv**	0	**91%**
FADpG		yes	yes	6 equiv	**3 equiv**	88%	**93%**
Glc-UDP	GlcppU-RNA	no	no	-	-	0	0
GlcppUpG		yes	no	**9 equiv**	-	**44%**	0
NAcGlc-UDP	NAcGlcppU-RNA	yes	no	-	-	0	0
NAcGlcppUpG		no	no	**9 equiv**	-	**48%**	0
pppUpG	pppU-RNA	yes	no	**9 equiv**	-	**54%**	0

The bold values correspond to the conditions (compound excess/promoter) affording the highest capping efficiencies for the given compound.

a φ 6.5 sequence: CAGTAA TACGAC TCACTA, TA G GGG, AAGCGG GCATGC GGCCAG CCATAG CCGATC A.

b φ 2.5 sequence: CAGTAA, TACGAC TCACTA TT A GGG, AAGCGG GCATGC GGCCAG CCATAG CCGATC A.

## Data Availability

The original contributions presented in the study are included in the article/Supplementary Material, further inquiries can be directed to the corresponding author.

## References

[B1] Abele F. Hofer K. Bernhard P. Grawenhoff J. Seidel M. Krause A. (2020). A novel NAD-RNA decapping pathway discovered by synthetic light-up NAD-RNAs. Biomolecules 10, 513. 10.3390/biom10040513 PMC7226252 32231086

[B2] Beckert B. Masquida B. (2011). Synthesis of RNA by *in vitro* transcription. Methods Mol. Biol. 703, 29–41. 10.1007/978-1-59745-248-9_3 21125481

[B3] Benoni R. Culka M. Hudecek O. Gahurova L. Cahová H. (2020). Dinucleoside polyphosphates as RNA building blocks with pairing ability in transcription initiation. ACS Chem. Biol. 15, 1765–1772. 10.1021/acschembio.0c00178 32530599

[B4] Birdsall R. E. Gilar M. Shion H. Yu Y. Q. Chen W. (2016). Reduction of metal adducts in oligonucleotide mass spectra in ion-pair reversed-phase chromatography/mass spectrometry analysis. Rapid Commun. Mass Spectrom. 30, 1667–1679. 10.1002/rcm.7596 28328039 PMC5094505

[B5] Cahová H. Winz M. L. Hofer K. Nubel G. Jaschke A. (2015). NAD captureSeq indicates NAD as a bacterial cap for a subset of regulatory RNAs. Nature 519, 374–377. 10.1038/nature14020 25533955

[B6] Chen Y. G. Kowtoniuk W. E. Agarwal I. Shen Y. Liu D. R. (2009). LC/MS analysis of cellular RNA reveals NAD-linked RNA. Nat. Chem. Biol. 5, 879–881. 10.1038/nchembio.235 19820715 PMC2842606

[B7] Coleman T. M. Wang G. Huang F. (2004). Superior 5' homogeneity of RNA from ATP-initiated transcription under the T7 phi 2.5 promoter. Nucleic Acids Res. 32, e14. 10.1093/nar/gnh007 14744982 PMC373309

[B8] Dabrowski-Tumanski P. Kowalska J. Jemielity J. (2013). Efficient and rapid synthesis of nucleoside diphosphate sugars from nucleoside phosphorimidazolides. Eur. J. Org. Chem. 2013, 2147–2154. 10.1002/ejoc.201201466

[B9] Depaix A. Mlynarska-Cieslak A. Warminski M. Sikorski P. J. Jemielity J. Kowalska J. (2021). RNA ligation for mono and dually labeled RNAs. Chemistry 27, 12190–12197. 10.1002/chem.202101909 34114681

[B10] Doamekpor S. K. Grudzien-Nogalska E. Mlynarska-Cieslak A. Kowalska J. Kiledjian M. Tong L. (2020). DXO/Rai1 enzymes remove 5'-end FAD and dephospho-CoA caps on RNAs. Nucleic Acids Res. 48, 6136–6148. 10.1093/nar/gkaa297 32374864 PMC7293010

[B11] Fuller D. H. Berglund P. (2020). Amplifying RNA vaccine development. N. Engl. J. Med. 382, 2469–2471. 10.1056/NEJMcibr2009737 32558474

[B12] Furuichi Y. Muthukrishnan S. Shatkin A. J. (1975). 5'-Terminal m-7G(5')ppp(5')G-m-p *in vivo*: Identification in reovirus genome RNA. Proc. Natl. Acad. Sci. U. S. A. 72, 742–745. 10.1073/pnas.72.2.742 1054852 PMC432392

[B13] Furuichi Y. Shatkin A. J. (2000). Viral and cellular mRNA capping: Past and prospects. Adv. Virus Res. 55, 135–184. 10.1016/s0065-3527(00)55003-9 11050942 PMC7131690

[B14] Grudzien-Nogalska E. Stepinski J. Jemielity J. Zuberek J. Stolarski R. Rhoads R. E. (2007). Synthesis of anti-reverse cap analogs (ARCAs) and their applications in mRNA translation and stability. Methods Enzymol. 431, 203–227. 10.1016/S0076-6879(07)31011-2 17923237

[B15] Helm M. Brule H. Giege R. Florentz C. (1999). More mistakes by T7 RNA polymerase at the 5' ends of *in vitro*-transcribed RNAs. RNA 5, 618–621. 10.1017/s1355838299982328 10334331 PMC1369788

[B16] Huang F. (2003). Efficient incorporation of CoA, NAD and FAD into RNA by *in vitro* transcription. Nucleic Acids Res. 31, e8. 10.1093/nar/gng008 12560511 PMC149220

[B17] Huang H. Weng H. Chen J. (2020). The biogenesis and precise control of RNA m(6)A methylation. Trends Genet. 36, 44–52. 10.1016/j.tig.2019.10.011 31810533 PMC6925345

[B18] Hudecek O. Benoni R. Reyes-Gutierrez P. E. Culka M. Sanderova H. Hubalek M. (2020). Dinucleoside polyphosphates act as 5'-RNA caps in bacteria. Nat. Commun. 11, 1052. 10.1038/s41467-020-14896-8 32103016 PMC7044304

[B19] Ishikawa M. Murai R. Hagiwara H. Hoshino T. Suyama K. (2009). Preparation of eukaryotic mRNA having differently methylated adenosine at the 5'-terminus and the effect of the methyl group in translation. Nucleic Acids Symp. Ser. (Oxf). 129–130. 10.1093/nass/nrp065 19749294

[B20] Jemielity J. Fowler T. Zuberek J. Stepinski J. Lewdorowicz M. Niedzwiecka A. (2003). Novel "anti-reverse" cap analogs with superior translational properties. RNA 9, 1108–1122. 10.1261/rna.5430403 12923259 PMC1370475

[B21] Jia L. Qian S. B. (2021). Therapeutic mRNA engineering from head to tail. Acc. Chem. Res. 54, 4272–4282. 10.1021/acs.accounts.1c00541 34756012

[B22] Julius C. Salgado P. S. Yuzenkova Y. (2020). Metabolic cofactors NADH and FAD act as non-canonical initiating substrates for a primase and affect replication primer processing *in vitro* . Nucleic Acids Res. 48, 7298–7306. 10.1093/nar/gkaa447 32463447 PMC7367122

[B23] Julius C. Yuzenkova Y. (2017). Bacterial RNA polymerase caps RNA with various cofactors and cell wall precursors. Nucleic Acids Res. 45, 8282–8290. 10.1093/nar/gkx452 28531287 PMC5737558

[B24] Kowtoniuk W. E. Shen Y. Heemstra J. M. Agarwal I. Liu D. R. (2009). A chemical screen for biological small molecule-RNA conjugates reveals CoA-linked RNA. Proc. Natl. Acad. Sci. U. S. A. 106, 7768–7773. 10.1073/pnas.0900528106 19416889 PMC2674394

[B25] Levin D. S. Shepperd B. T. Gruenloh C. J. (2011). Combining ion pairing agents for enhanced analysis of oligonucleotide therapeutics by reversed phase-ion pairing ultra performance liquid chromatography (UPLC). J. Chromatogr. B Anal. Technol. Biomed. Life Sci. 879, 1587–1595. 10.1016/j.jchromb.2011.03.051 21514903

[B26] Luciano D. J. Levenson-Palmer R. Belasco J. G. (2019). Stresses that raise Np4A levels induce protective nucleoside tetraphosphate capping of bacterial RNA. Mol. Cell 75, 957–966. 10.1016/j.molcel.2019.05.031 31178354 PMC6731127

[B27] Mattay J. Dittmar M. Rentmeister A. (2021). Chemoenzymatic strategies for RNA modification and labeling. Curr. Opin. Chem. Biol. 63, 46–56. 10.1016/j.cbpa.2021.01.008 33690011

[B28] Milligan J. F. Groebe D. R. Witherell G. W. Uhlenbeck O. C. (1987). Oligoribonucleotide synthesis using T7 RNA polymerase and synthetic DNA templates. Nucleic Acids Res. 15, 8783–8798. 10.1093/nar/15.21.8783 3684574 PMC306405

[B29] Mlynarska-Cieslak A. Depaix A. Grudzien-Nogalska E. Sikorski P. J. Warminski M. Kiledjian M. (2018). Nicotinamide-containing di- and trinucleotides as chemical tools for studies of NAD-capped RNAs. Org. Lett. 20, 7650–7655. 10.1021/acs.orglett.8b03386 30479128

[B30] Mohler M. Hofer K. Jaschke A. (2020). Synthesis of 5'-thiamine-capped RNA. Molecules 25, 5492. 10.3390/molecules25235492 PMC7727699 33255222

[B31] Netzband R. Pager C. T. (2020). Epitranscriptomic marks: Emerging modulators of RNA virus gene expression. Wiley Interdiscip. Rev. RNA 11, e1576. 10.1002/wrna.1576 31694072 PMC7169815

[B32] Nubel G. Sorgenfrei F. A. Jaschke A. (2017). Boronate affinity electrophoresis for the purification and analysis of cofactor-modified RNAs. Methods 117, 14–20. 10.1016/j.ymeth.2016.09.008 27645507

[B33] Pardi N. Hogan M. J. Weissman D. (2020). Recent advances in mRNA vaccine technology. Curr. Opin. Immunol. 65, 14–20. 10.1016/j.coi.2020.01.008 32244193

[B34] Pascolo S. (2021). Synthetic messenger RNA-based vaccines: From scorn to hype. Viruses 13, 270. 10.3390/v13020270 33572452 PMC7916233

[B35] Patiny L. Borel A. (2013). ChemCalc: A building block for tomorrow's chemical infrastructure. J. Chem. Inf. Model. 53, 1223–1228. 10.1021/ci300563h 23480664

[B36] Pleiss J. A. Derrick M. L. Uhlenbeck O. C. (1998). T7 RNA polymerase produces 5' end heterogeneity during *in vitro* transcription from certain templates. RNA 4, 1313–1317. 10.1017/s135583829800106x 9769105 PMC1369703

[B37] Ren X. Deng R. Zhang K. Sun Y. Li Y. Li J. (2021). Single-Cell imaging of m(6) A modified RNA using m(6) A-specific *in situ* hybridization mediated proximity ligation assay (m(6) AISH-PLA). Angew. Chem. Int. Ed. Engl. 60, 22646–22651. 10.1002/anie.202109118 34291539

[B38] Sato Y. Iwasawa D. Hui K. P. Nakagomi R. Nishizawa S. (2018). Improved boronate affinity electrophoresis by optimization of the running buffer for a single-step separation of piRNA from mouse testis total RNA. Anal. Sci. 34, 627–630. 10.2116/analsci.17N024 29743438

[B39] Schubert S. Gul D. C. Grunert H. P. Zeichhardt H. Erdmann V. A. Kurreck J. (2003). RNA cleaving '10-23' DNAzymes with enhanced stability and activity. Nucleic Acids Res. 31, 5982–5992. 10.1093/nar/gkg791 14530446 PMC219472

[B40] Schurer H. Lang K. Schuster J. Morl M. (2002). A universal method to produce *in vitro* transcripts with homogeneous 3' ends. Nucleic Acids Res. 30, e56. 10.1093/nar/gnf055 12060694 PMC117298

[B41] Sikorski P. J. Warminski M. Kubacka D. Ratajczak T. Nowis D. Kowalska J. (2020). The identity and methylation status of the first transcribed nucleotide in eukaryotic mRNA 5' cap modulates protein expression in living cells. Nucleic Acids Res. 48, 1607–1626. 10.1093/nar/gkaa032 31984425 PMC7038993

[B42] Strohalm M. Kavan D. Novak P. Volny M. Havlicek V. (2010). mMass 3: a cross-platform software environment for precise analysis of mass spectrometric data. Anal. Chem. 82, 4648–4651. 10.1021/ac100818g 20465224

[B43] Wang J. Alvin Chew B. L. Lai Y. Dong H. Xu L. Balamkundu S. (2019). Quantifying the RNA cap epitranscriptome reveals novel caps in cellular and viral RNA. Nucleic Acids Res. 47, e130. 10.1093/nar/gkz751 31504804 PMC6847653

[B44] Wang Q. Weng L. Jiang H. Zhang S. Toyoda T. (2013). Fluorescent primer-based *in vitro* transcription system of viral RNA-dependent RNA polymerases. Anal. Biochem. 433, 92–94. 10.1016/j.ab.2012.10.025 23103398

[B45] Wei C. M. Gershowitz A. Moss B. (1975). Methylated nucleotides block 5' terminus of HeLa cell messenger RNA. Cell 4, 379–386. 10.1016/0092-8674(75)90158-0 164293

[B46] Wiedermannova J. Julius C. Yuzenkova Y. (2021). The expanding field of non-canonical RNA capping: New enzymes and mechanisms. R. Soc. Open Sci. 8, 201979. 10.1098/rsos.201979 34017598 PMC8131947

[B47] Zhao L. Y. Song J. Liu Y. Song C. X. Yi C. (2020). Mapping the epigenetic modifications of DNA and RNA. Protein Cell 11, 792–808. 10.1007/s13238-020-00733-7 32440736 PMC7647981

