# Supplementary material for: Preparation of RNAs with non-canonical 5′ ends using novel di- and trinucleotide reagents for co-transcriptional capping
Source: Front Mol Biosci. 2022 Aug 19;9:854170. doi: 10.3389/fmolb.2022.854170 (PMC9437278; doi:10.3389/fmolb.2022.854170)

Analytical data

Preparation of RNAs with non-canonical 5′ ends using novel di- and trinucleotide reagents for co-transcriptional capping

Anaïs Depaix,[1,2] Ewa Grudzien-Nogalska,[3] Bartlomiej Fedorczyk,[2] Megerditch Kiledjian,[3] Jacek Jemielity,[2]* Joanna Kowalska[1] *

[1] Division of Biophysics, Institute of Experimental Physics, Faculty of Physics, University of Warsaw, Warsaw, Poland

[2] Centre of New Technologies, University of Warsaw, Warsaw, Poland

[3] Department of Cell Biology and Neuroscience, Rutgers University, New York, NJ, United States

***Correspondence:**
Jacek Jemielity
j.jemielity@cent.uw.edu.pl

Joanna Kowalska
jkowalska@fuw.edu.pl

Table S1. Identification of various RNA variants present in the analyzed samples. Theoretical values were generated with the use of ChemCalc tool, experimental values were calculated by means of mass spectrum deconvolution.

	General Formula	RNA Type	Experimental [Da]	Theoretical [Da]	differance [Da]	Accuracy [ppm]
FAD	$C_{329}H_{395}N_{146}O_{239}P_{36}Na_{14}$	35ntRNA+GG-ACU	11656.69	11655.42	-1.27	-109.38
	$C_{328}H_{394}N_{143}O_{239}P_{36}Na_{14}$	35ntRNA-C	11601.83	11600.38	-1.45	-124.90
	$C_{338}H_{406}N_{148}O_{247}P_{37}Na_{14}$	35ntRNA+GG-AC	11961.99	11961.58	-0.41	-34.05
	$C_{337}H_{406}N_{146}O_{246}P_{37}Na_{14}$	35ntRNA	11906.44	11905.56	-0.88	-73.63
	$C_{346}H_{412}N_{149}O_{240}P_{35}Na_{13}$	FADRNA-2C/2U	11879.25	11880.79	1.54	129.29
	$C_{355}H_{424}N_{152}O_{247}P_{36}Na_{13}$	FADRNA-C/U	12184.95	12185.97	1.02	83.63
	$C_{356}H_{425}N_{155}O_{246}P_{36}Na_{13}$	FADRNA+G-CU	12223.36	12225.01	1.65	135.05
	$C_{365}H_{436}N_{157}O_{254}P_{37}Na_{13}$	FADRNA-G+C	12529.40	12531.18	1.78	142.22
	$C_{356}H_{425}N_{155}O_{246}P_{36}Na_{13}$	FADRNA-G+CU	12229.18	12225.01	-4.17	-340.74
	$C_{356}H_{425}N_{155}O_{245}P_{36}Na_{13}$	FADRNA-A+CU	12207.52	12209.01	1.49	122.15
	General Formula	RNA Type	Experimental [Da]	Theoretical [Da]	differance [Da]	Accuracy [ppm]
FADpG	$C_{338}H_{406}N_{148}O_{247}P_{37}Na_{14}$	35ntRNA+GG-AC	11960.56	11961.58	1.02	85.22
	$C_{337}H_{406}N_{146}O_{246}P_{37}Na_{14}$	35ntRNA	11904.90	11905.56	0.66	55.35
	$C_{347}H_{412}N_{151}O_{241}P_{35}Na_{13}$	FADRNA+GG-ACCC	11935.16	11936.81	1.65	138.57
	$C_{346}H_{412}N_{149}O_{240}P_{35}Na_{13}$	FADRNA-2C/2U	11879.33	11880.79	1.46	123.03
	$C_{356}H_{424}N_{154}O_{248}P_{36}Na_{13}$	FADRNA+GG-ACC	12240.62	12242.00	1.38	112.76
	$C_{355}H_{424}N_{152}O_{247}P_{36}Na_{13}$	FADRNA-C/U	12184.99	12185.97	0.98	80.51
	$C_{364}H_{436}N_{155}O_{254}P_{37}Na_{13}$	FADRNA	12490.39	12491.15	0.76	61.24
	$C_{365}H_{436}N_{157}O_{254}P_{37}Na_{13}$	FADRNA-G+C	12530.28	12531.18	0.90	71.54
	$C_{356}H_{425}N_{155}O_{245}P_{36}Na_{13}$	FADRNA-A+CU	12208.41	12209.01	0.60	49.13
	$C_{373}H_{448}N_{158}O_{261}P_{38}Na_{13}$	FADRNA-C/U	12794.61	12796.34	1.72	134.65
	General Formula	RNA Type	Experimental [Da]	Theoretical [Da]	differance [Da]	Accuracy [ppm]
Glc	$C_{343}H_{414}N_{145}O_{250}P_{36}Na_{14}$	GlcRNA-C/U	12005.08	12004.71	-0.38	-31.43
	$C_{352}H_{426}N_{148}O_{257}P_{37}Na_{14}$	GlcRNA	12311.47	12309.89	-1.58	-128.34
	$C_{353}H_{426}N_{150}O_{257}P_{37}Na_{14}$	GlcRNA-G+C	12349.50	12349.91	0.41	33.56
	$C_{361}H_{438}N_{151}O_{264}P_{38}Na_{14}$	GlcRNA+C	12617.62	12615.07	-2.55	-202.21
	$C_{362}H_{438}N_{153}O_{264}P_{38}Na_{14}$	GlcRNA+G	12656.54	12655.09	-1.45	-114.51
	$C_{353}H_{427}N_{151}O_{255}P_{37}Na_{14}$	GlcRNA+A-U	12335.02	12332.93	-2.09	-169.43
	$C_{362}H_{438}N_{153}O_{263}P_{38}Na_{14}$	GlcRNA+A	12639.64	12639.09	-0.55	-43.24
	$C_{327}H_{393}N_{141}O_{241}P_{36}Na_{15}$	35ntRNA-A	11615.82	11614.34	-1.48	-127.50
	$C_{329}H_{394}N_{146}O_{239}P_{36}Na_{15}$	35ntRNA+G-CU	11672.01	11677.40	5.39	461.42
	$C_{337}H_{405}N_{146}O_{246}P_{37}Na_{15}$	35ntRNA+A-G	11923.19	11927.54	4.35	364.68
	$C_{338}H_{405}N_{148}O_{247}P_{37}Na_{15}$	35ntRNA+G-C	11978.31	11983.57	5.26	438.70
	$C_{337}H_{404}N_{145}O_{248}P_{37}Na_{15}$	35ntRNA+U-C	11944.66	11944.53	-0.13	-11.24
	$C_{339}H_{406}N_{151}O_{245}P_{37}Na_{15}$	35ntRNA+AG-CU	12001.49	12006.61	5.12	426.44
	$C_{346}H_{417}N_{149}O_{254}P_{38}Na_{15}$	35ntRNA+C	12251.11	12248.72	-2.39	-194.74
	$C_{347}H_{417}N_{151}O_{255}P_{38}Na_{15}$	35ntRNA+GG-A	12306.44	12304.75	-1.69	-137.27

NAcGlc

General Formula	RNA Type	Experimental [Da]	Theoretical [Da]	difference [Da]	Accuracy [ppm]
$C_{346}H_{418}N_{149}O_{248}P_{36}Na_{14}$	NacGlc-RNA+A-CU	12067.54	12068.80	1.26	104.36
$C_{345}H_{417}N_{146}O_{250}P_{36}Na_{14}$	NacGlc-RNA-C	12046.35	12045.76	-0.59	-48.96
$C_{354}H_{429}N_{149}O_{257}P_{37}Na_{14}$	NacGlc-RNA	12352.22	12350.94	-1.29	-104.08
$C_{355}H_{429}N_{151}O_{257}P_{37}Na_{14}$	NacGlc-RNA+G-C	12391.12	12390.96	-0.16	-12.70
$C_{355}H_{430}N_{152}O_{255}P_{37}Na_{14}$	NacGlc-RNA+A-U	12375.44	12373.98	-1.46	-117.68
$C_{364}H_{441}N_{154}O_{263}P_{38}Na_{14}$	NacGlc-RNA+A	12681.30	12680.15	-1.16	-91.23
$C_{329}H_{394}N_{146}O_{239}P_{36}Na_{15}$	35ntRNA+G-CU	11672.46	11677.40	4.93	422.57
$C_{327}H_{393}N_{141}O_{241}P_{36}Na_{15}$	35ntRNA-A	11616.45	11614.34	-2.12	-182.37
$C_{336}H_{404}N_{143}O_{249}P_{37}Na_{15}$	35ntRNA+U-A	11921.86	11920.50	-1.36	-113.82
$C_{338}H_{405}N_{148}O_{247}P_{37}Na_{15}$	35ntRNA-G+C	11979.34	11983.57	4.23	352.97
$C_{347}H_{417}N_{151}O_{253}P_{38}Na_{15}$	35ntRNA+A	12267.33	12272.75	5.42	441.82
$C_{348}H_{417}N_{153}O_{254}P_{38}Na_{15}$	35ntRNA+GG-C	12325.37	12328.77	3.40	275.86
$C_{337}H_{405}N_{146}O_{247}P_{37}Na_{15}$	35ntRNA	11946.19	11943.54	-2.65	-221.51
$C_{339}H_{406}N_{151}O_{245}P_{37}Na_{15}$	35ntRNA+AG-CU	12001.39	12006.61	5.22	434.49
$C_{348}H_{417}N_{153}O_{253}P_{38}Na_{15}$	35ntRNA+AG-C	12307.28	12312.77	5.49	445.66
$C_{346}H_{417}N_{149}O_{254}P_{38}Na_{15}$	35ntRNA+C	12250.69	12248.72	-1.97	-160.63
				Mediane [ppm]	49.13

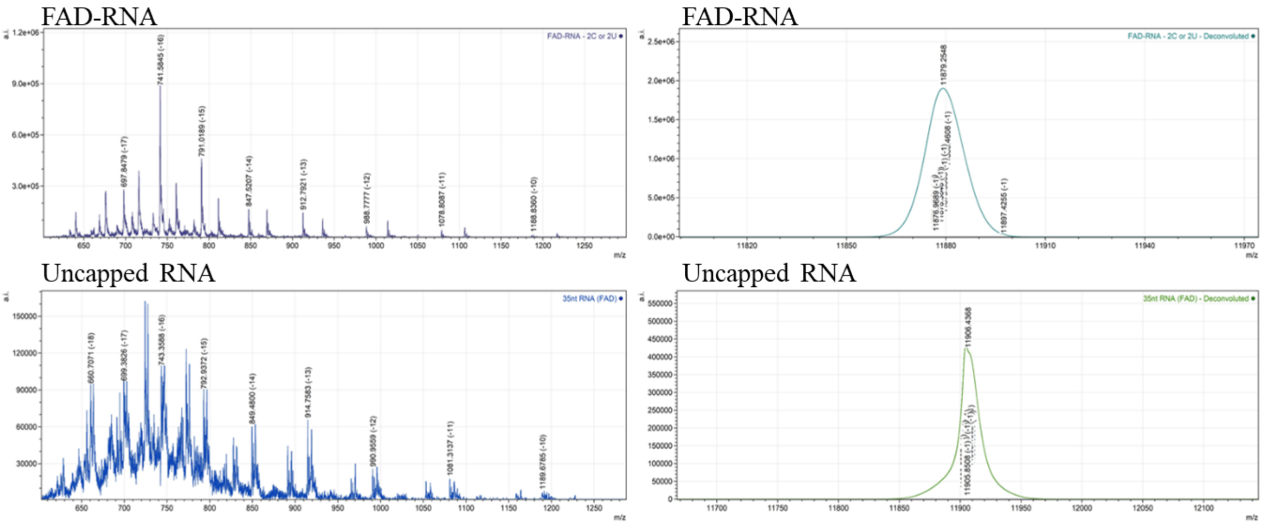

Figure S1. Respresentative spectrograms (left) and deconvoluted mass spectra (right) of FAD capped RNA - 2C/2U (up) and uncapped 35 nt RNA (down). Surprisingly, the major capped transcript present in the sample was two nucleotides shorter than expected. Net difference between C and U is 1 Da. It was impossible to distinguish those analogues with current resolution.

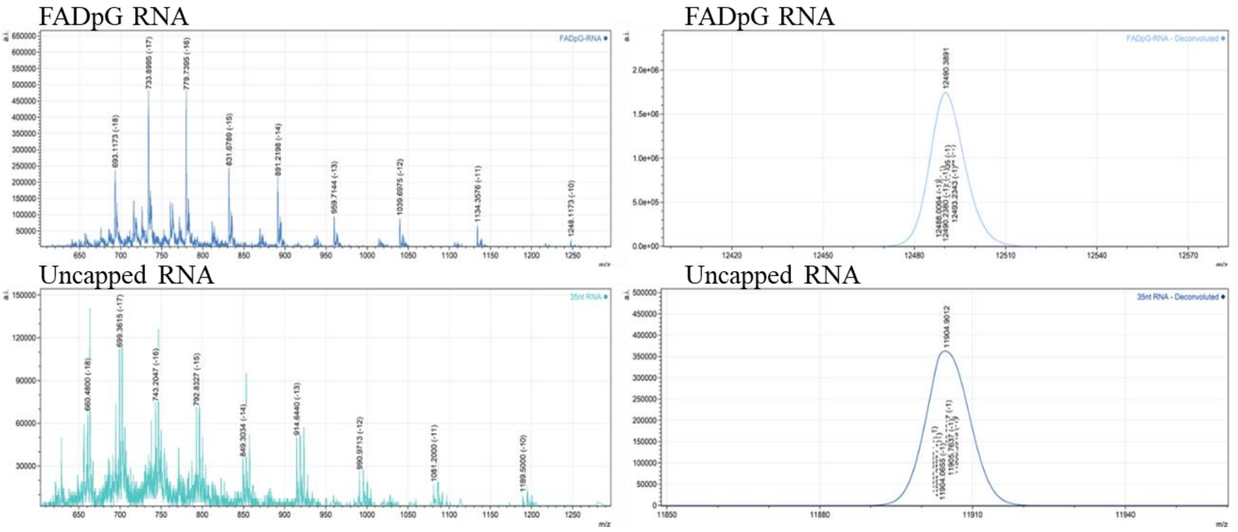

Figure S2. Representative spectrograms (left) and deconvoluted mass spectra (right) of FADpG initiated IVT RNA (up) and uncapped 35 nt RNA (down).

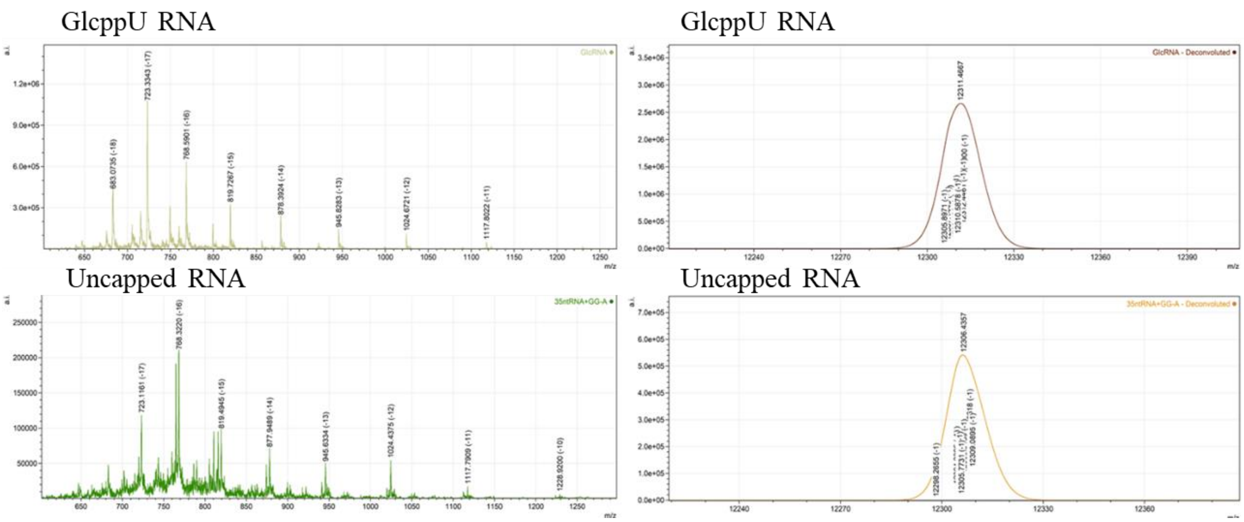

Figure S3. Representative spectrograms (left) and deconvoluted mass spectra (right) of GlcppU RNA (up) and uncapped 35 nt RNA (down). The major uncapped transcript present in the sample contained additional GG and deletion of A.

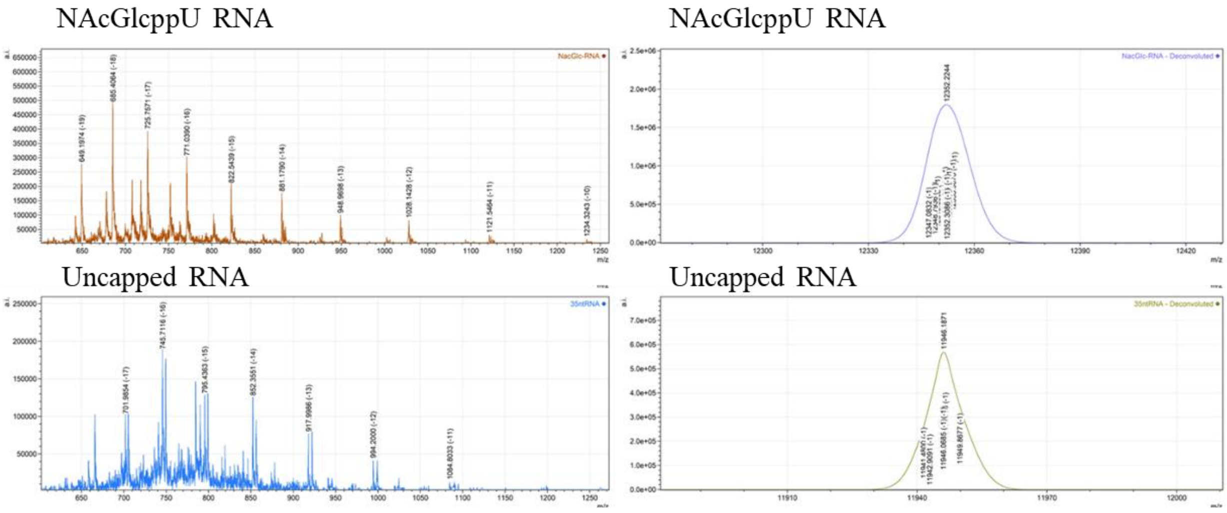

Figure S4. Representative spectrograms (left) and deconvoluted mass spectra (right) of NAcGlcppU RNA (up) and uncapped 35 nt RNA (down).

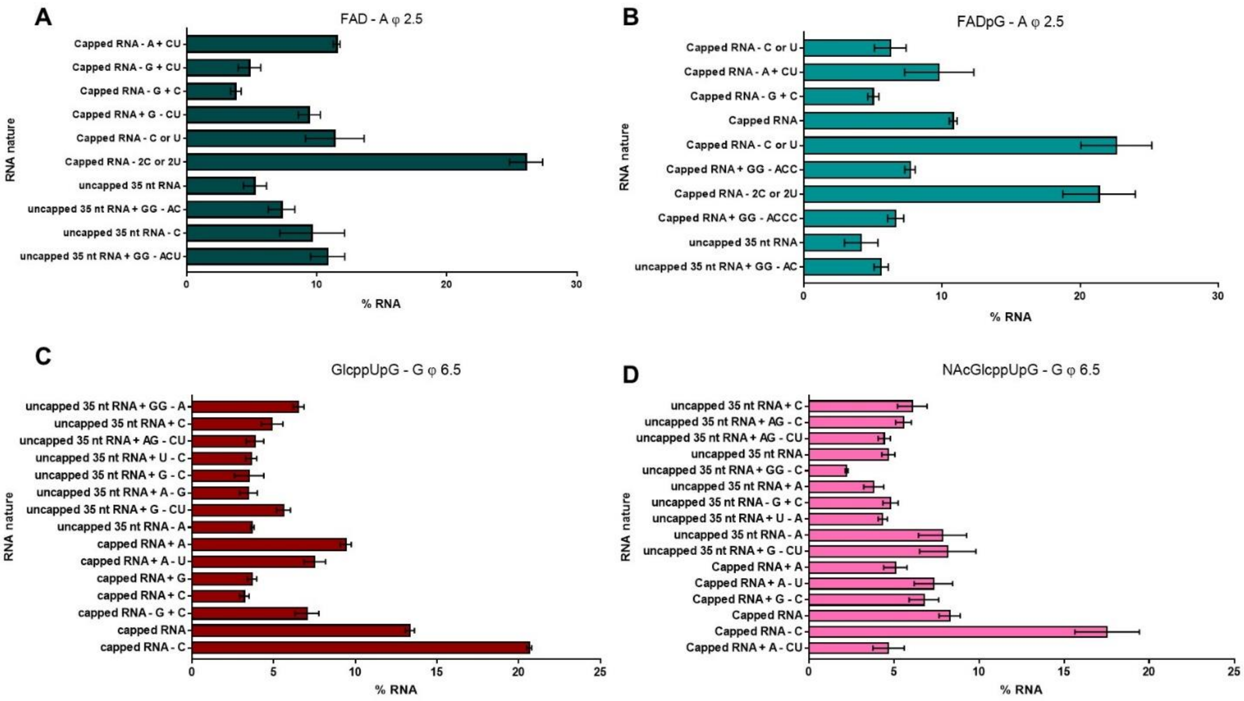

Figure S5. Abundance of the various RNA species found in the IVT samples analyzed by LC-MS method. (**A**) FAD initiated IVT RNA from A φ 2.5 template. (**B**) FADpG initiated IVT RNA from A φ 2.5 template. (**C**) GlcppUpG initiated IVT RNA from G φ 6.5 template. (**D**) NAcGlcppUpG initiated IVT RNA from G φ 6.5 template.

	FADpG (2)
Chemical structure	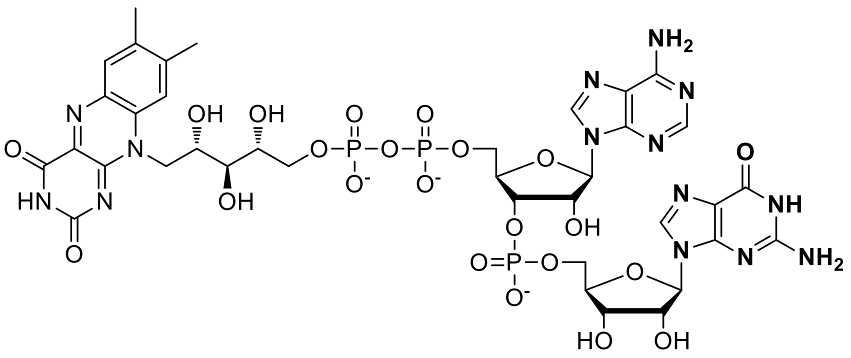
^{31}P NMR (162 MHz, D_2O)	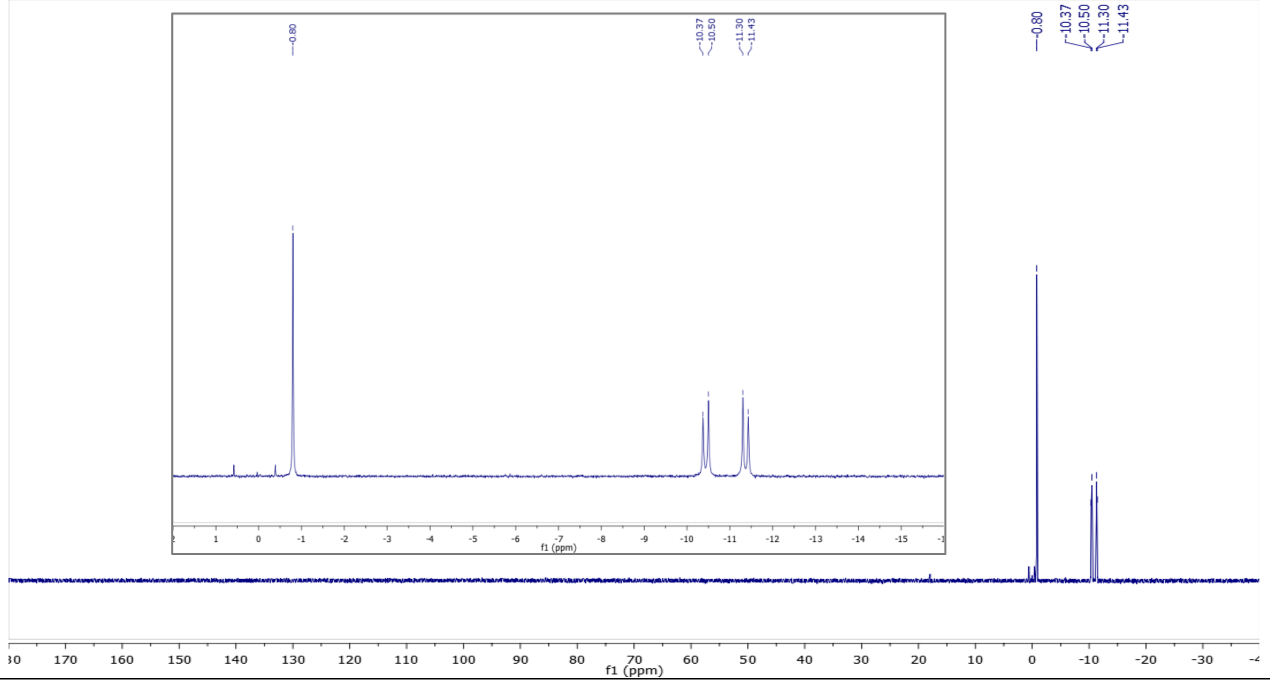

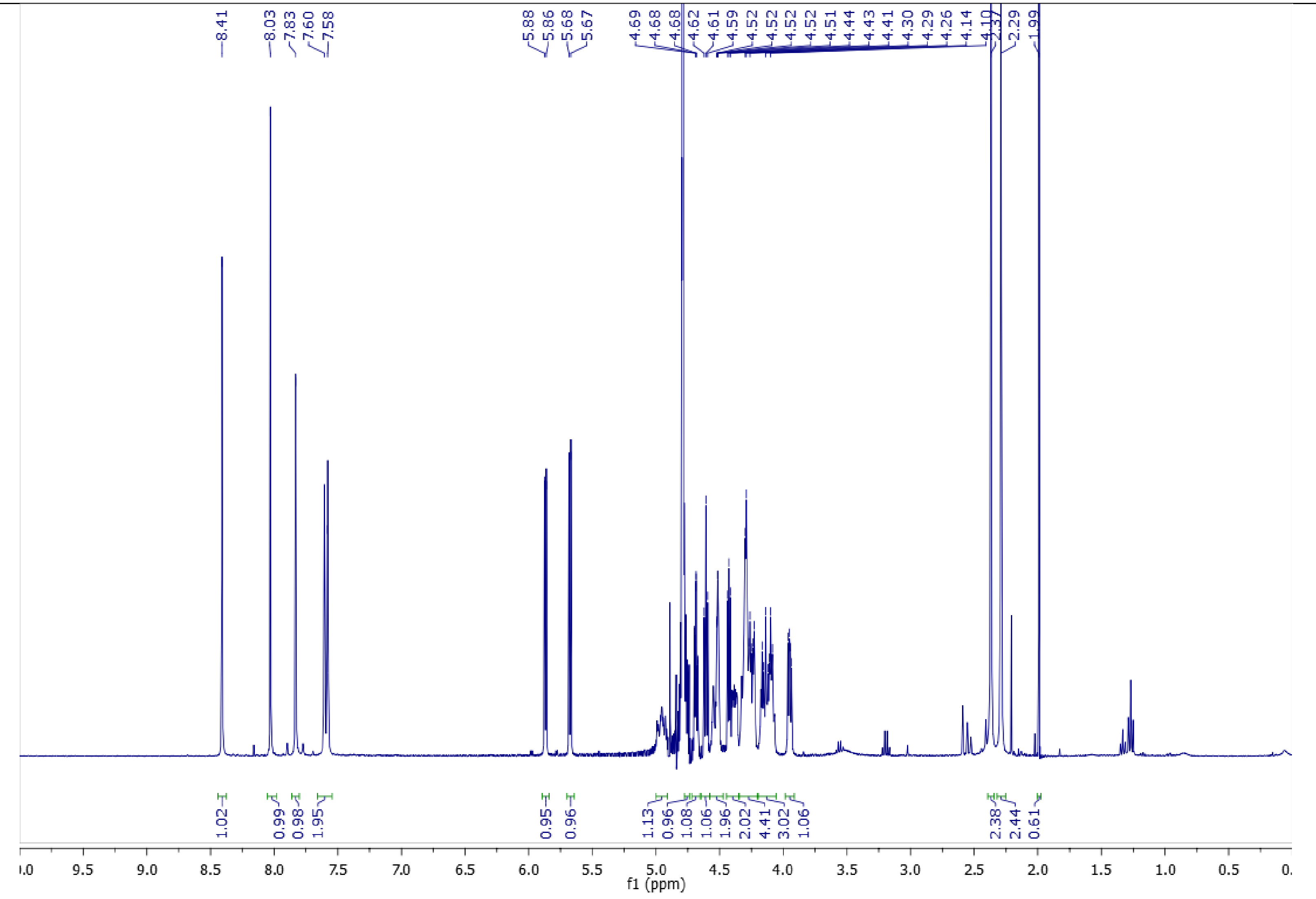

^{1}H NMR (400 MHz, D_2O)

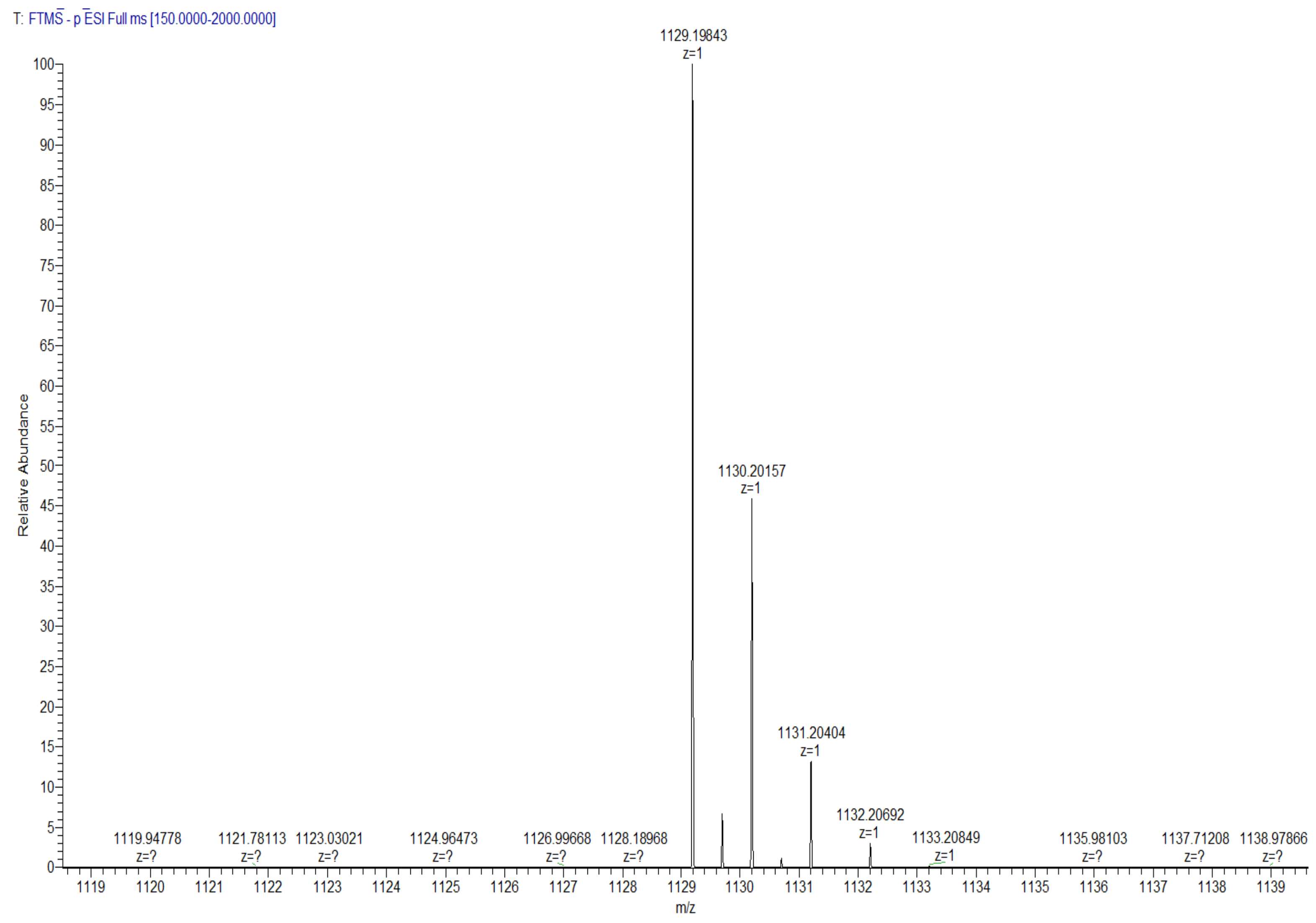

HRMS (-) ESI (Calc. [M-H]– $C_{37}H_{44}N_{14}O_{22}P_3^-$: 1129.19729)

	GlcppUpG (3)
Chemical structure	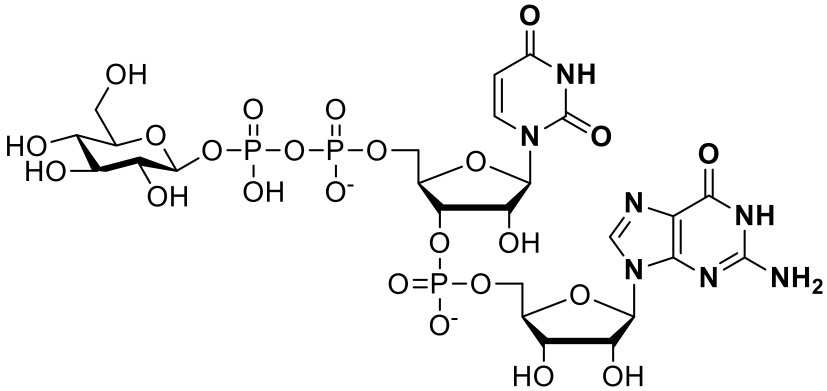
^{31}P NMR (203 MHz, D_2O)	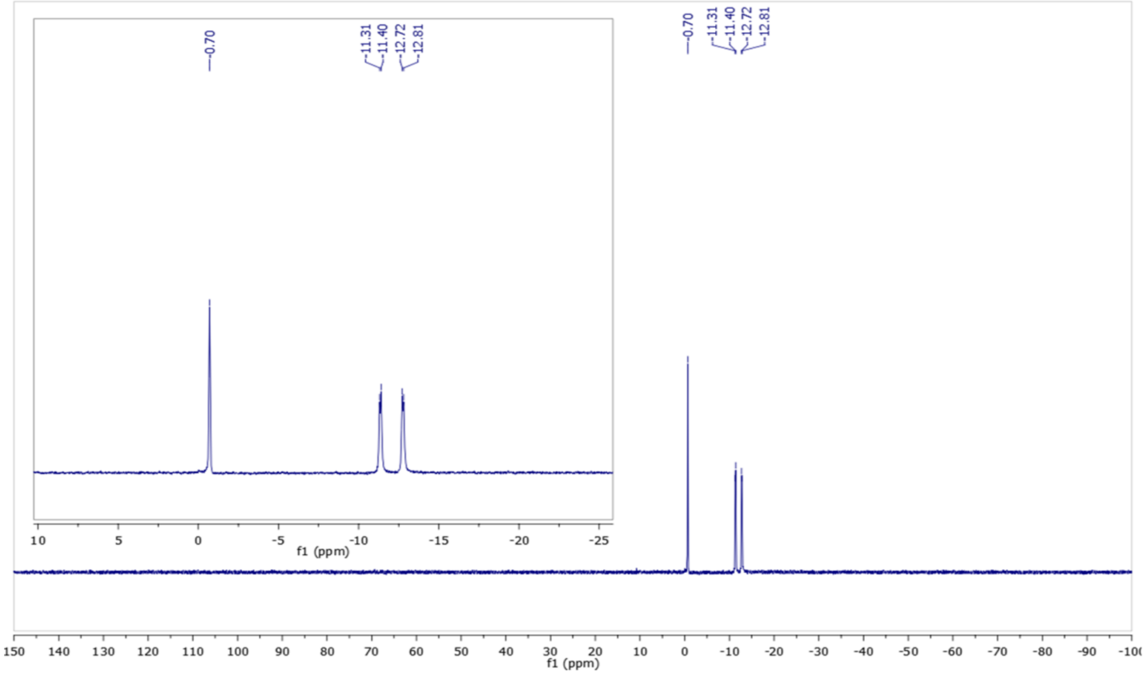

^{1}H NMR (500 MHz, D_2O)

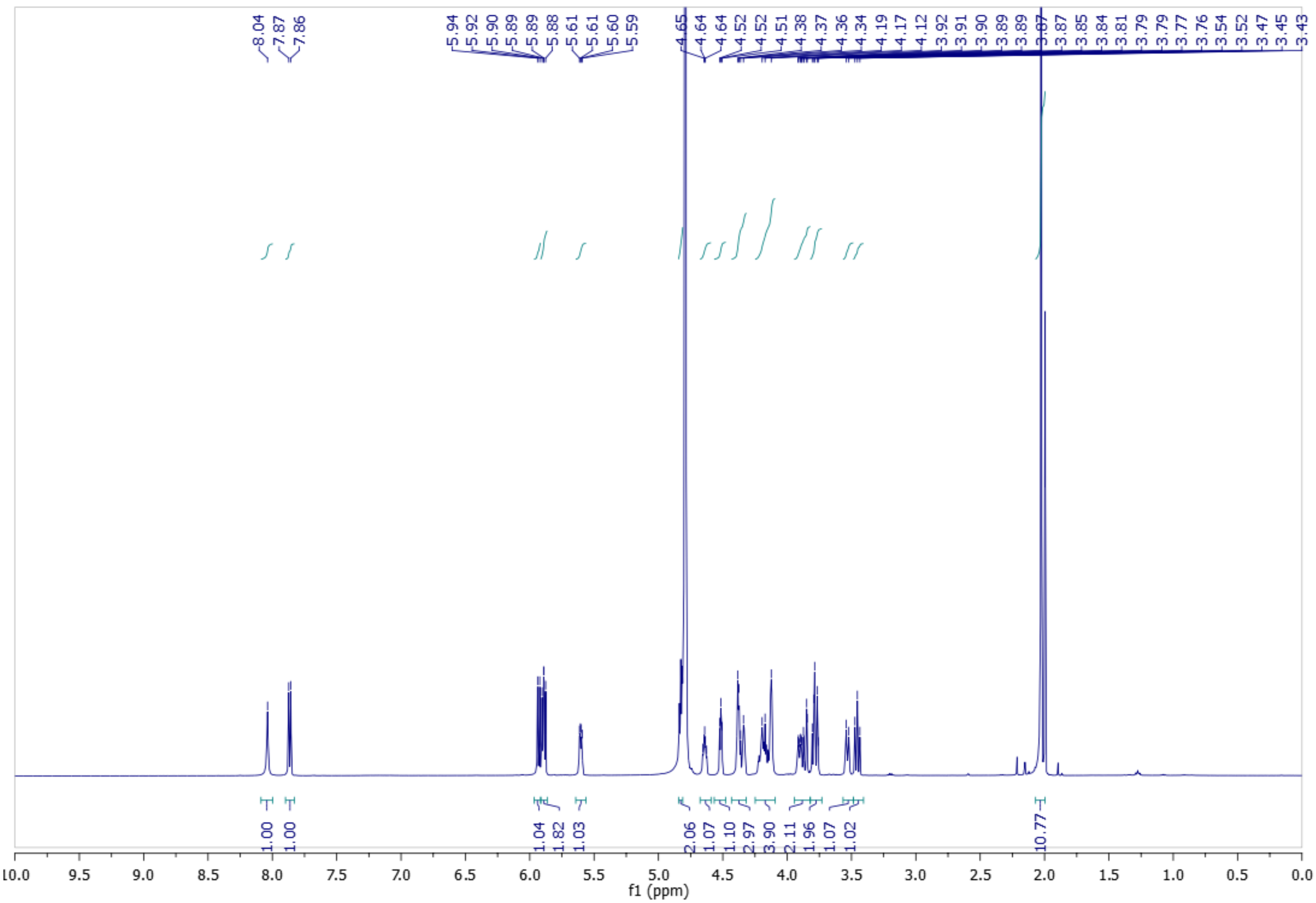

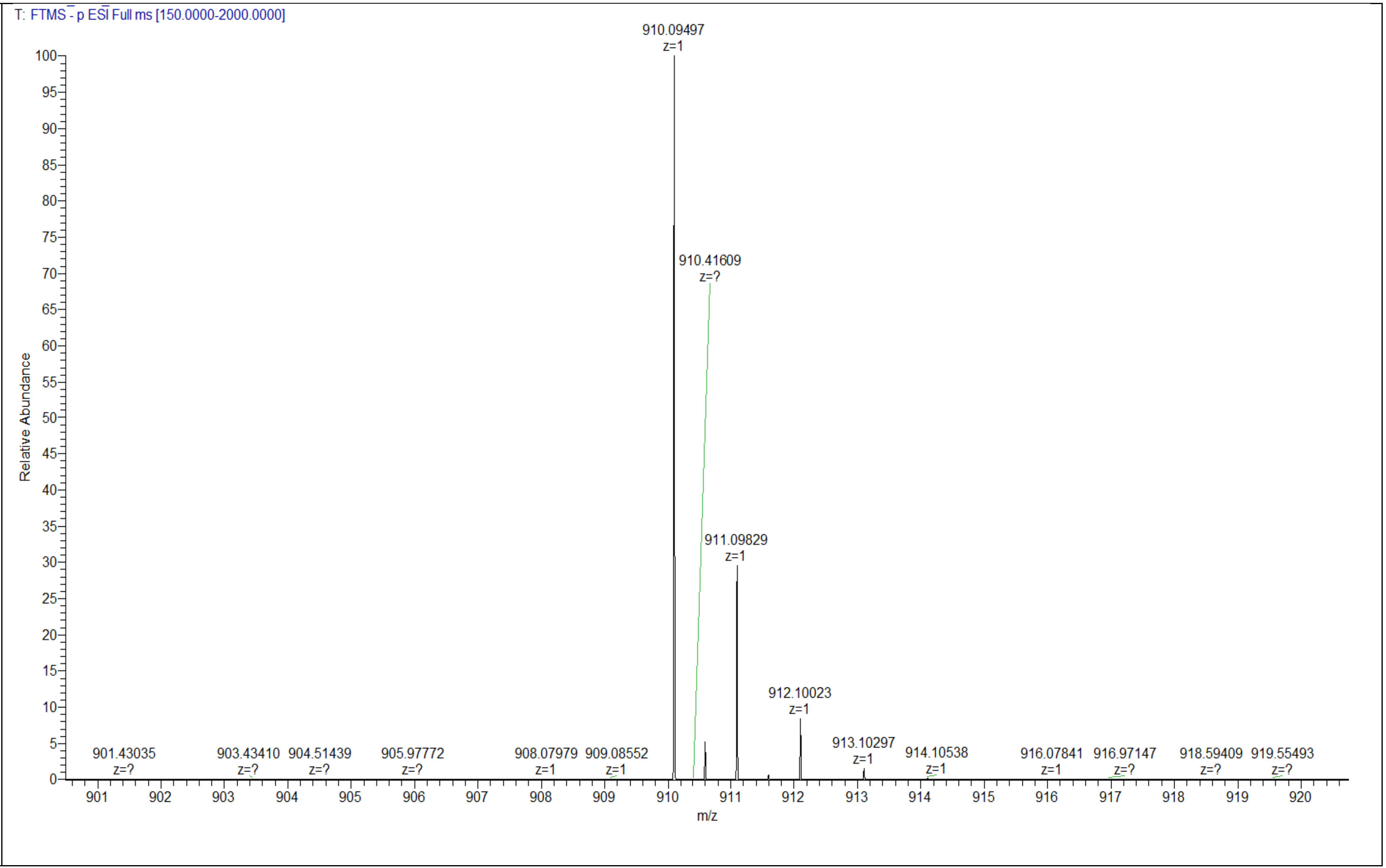

HRMS (-) ESI (Calc. [M-H]– $C_{25}H_{35}N_7O_{24}P_3$: 910..09518)

	NAcGlcppUpG (4)
Chemical structure	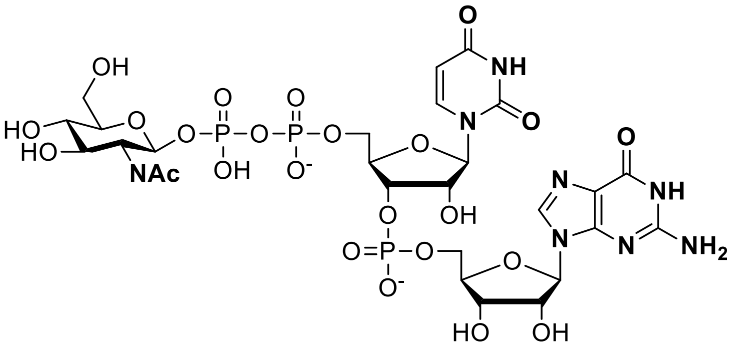
^{31}P NMR (162 MHz, D_2O)	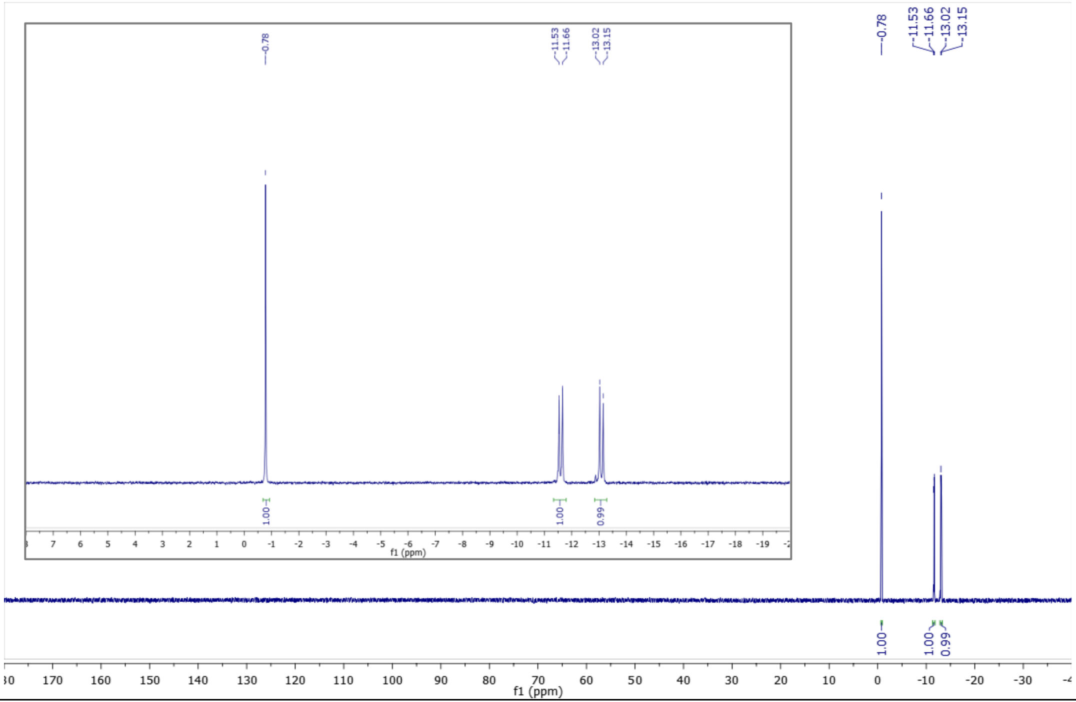

^1H NMR (400 MHz, D_2O)

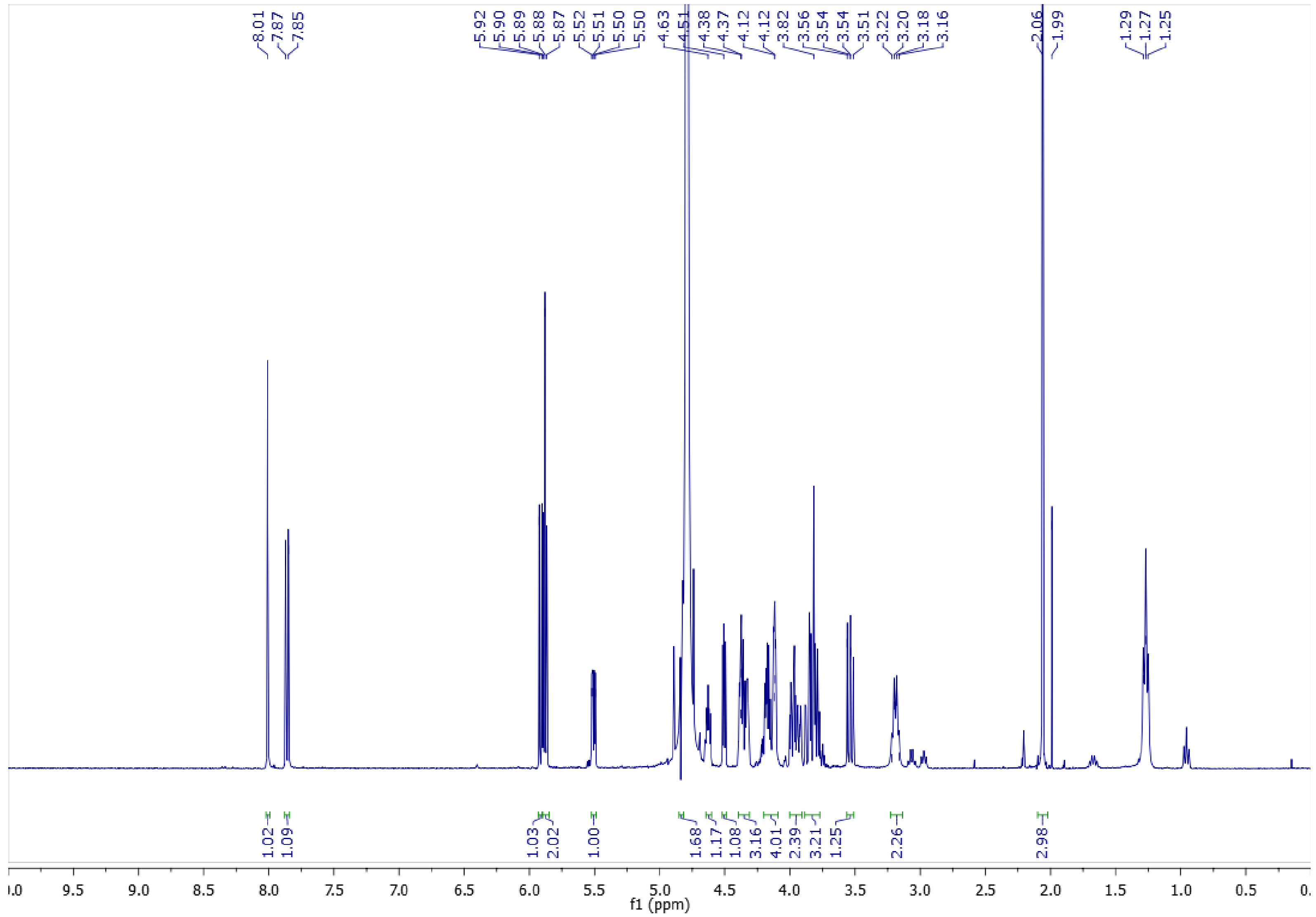

HRMS (-) ESI (Calc. [M-H]⁻ $C_{27}H_{38}N_8O_{24}P_3$: 951.012173)

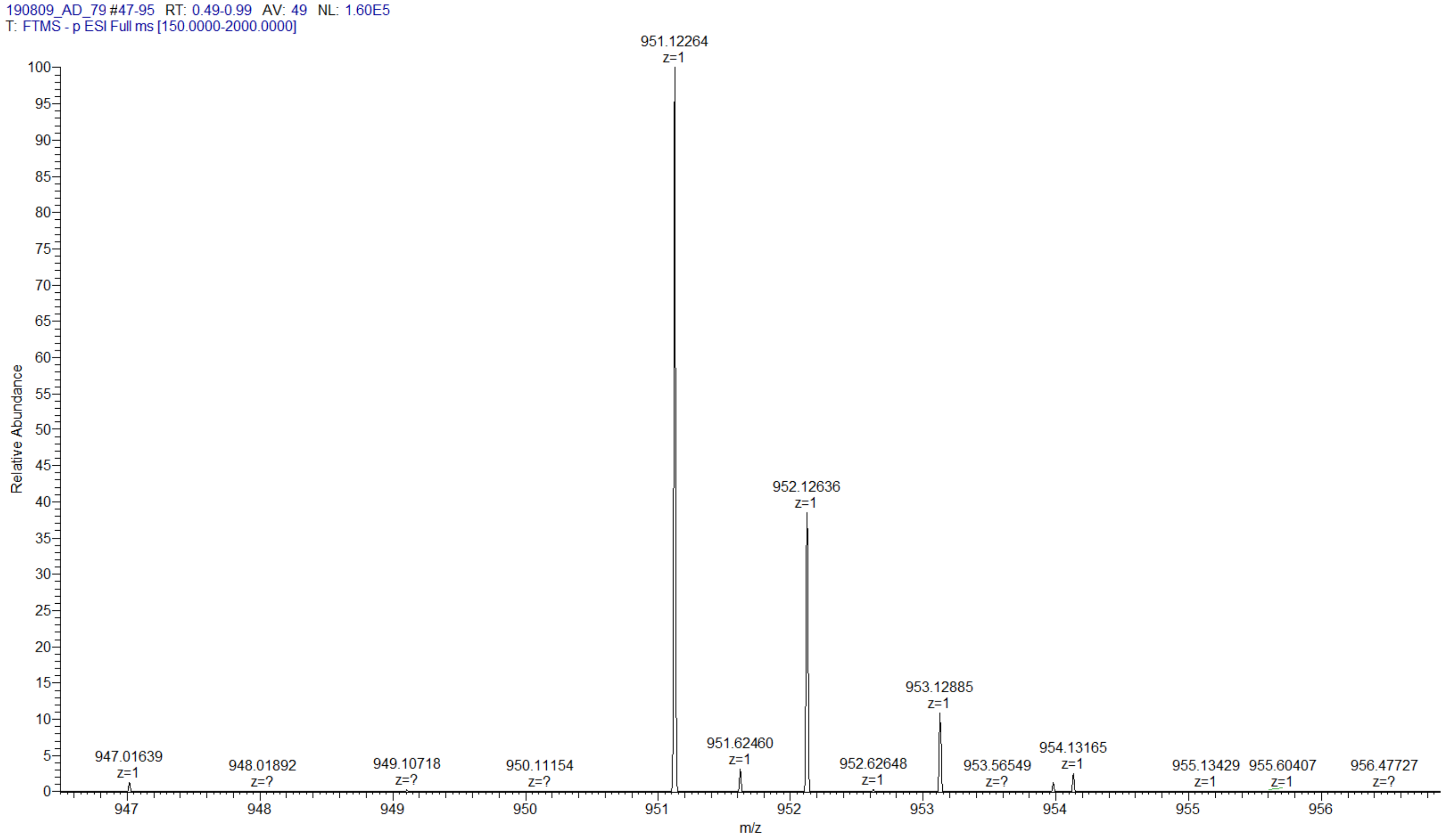

	**pppUpG (5)**
Chemical structure	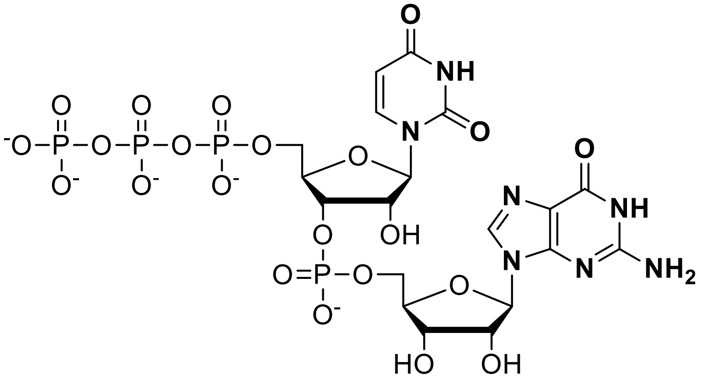
^{31}P NMR (162 MHz, D_2O)	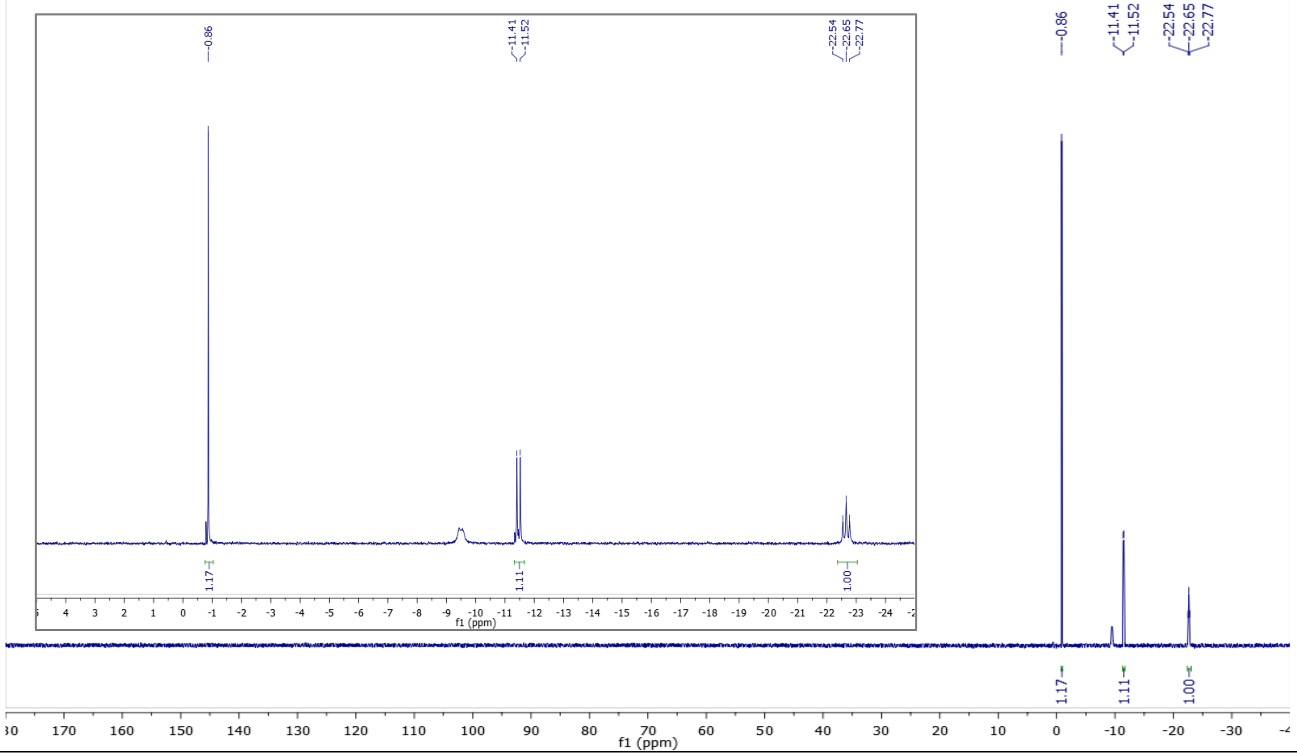

^1H NMR (400 MHz, D_2O)

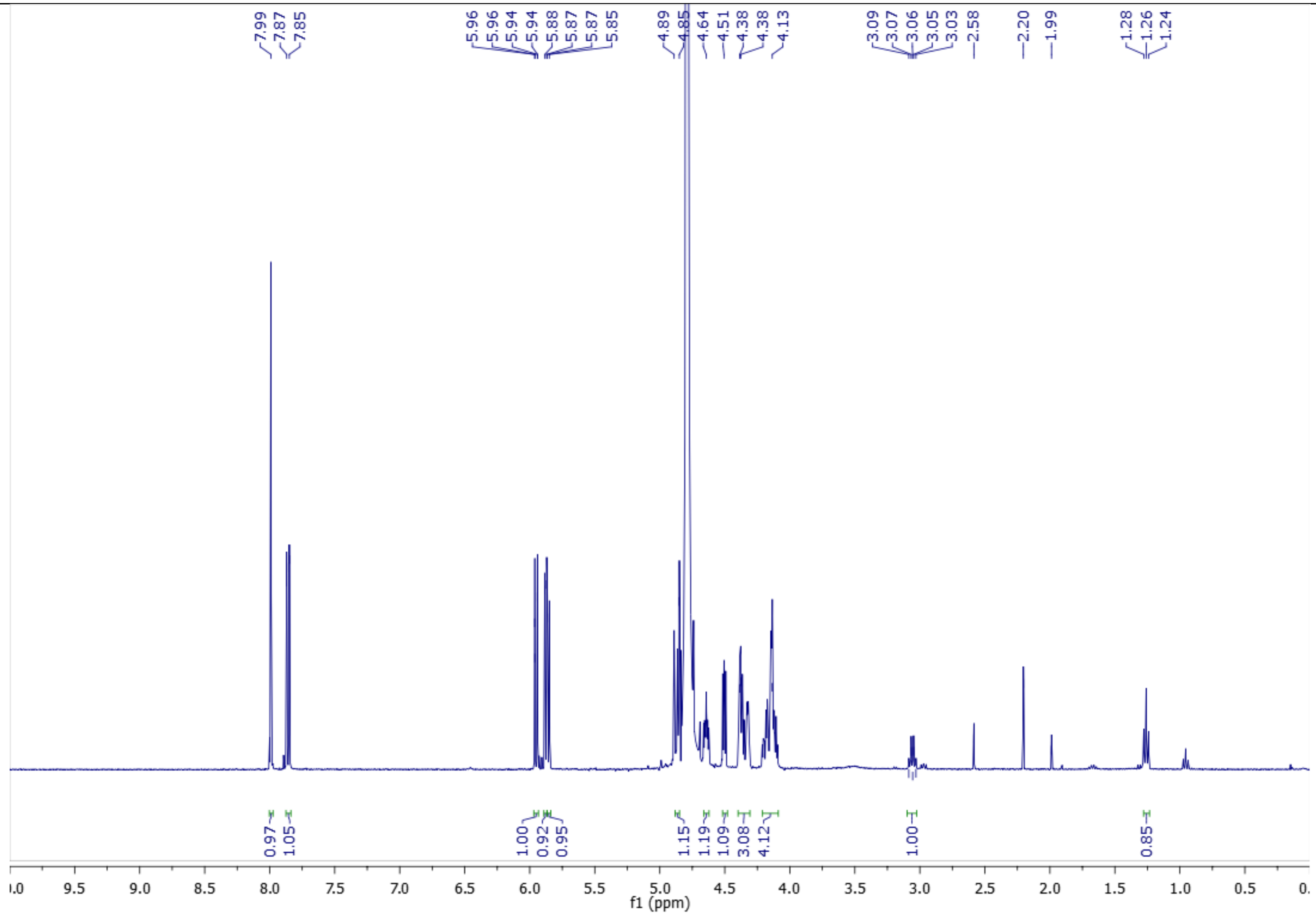

HRMS (-) ESI (Calc. $[M-H]^-$ $C_{19}H_{26}N_7O_{22}P_4$: 828.00869)

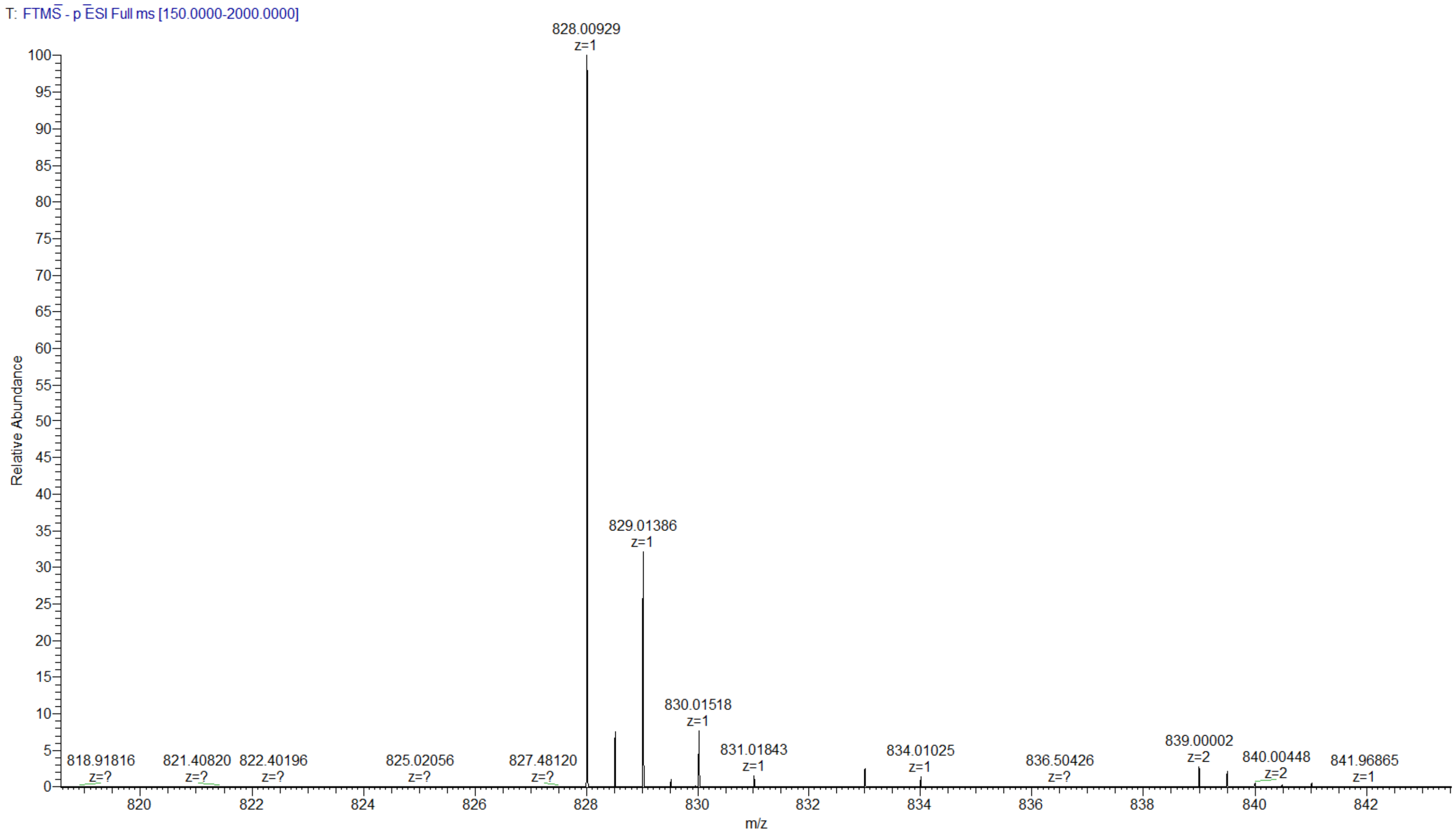

Supplement: Supplementary file 1 [file DataSheet1.pdf]
